# Senescence-Related Transcriptomic Analysis and Functional Characterization of ANGPTL4 in Lung Adenocarcinoma

**DOI:** 10.3390/cancers18213571

**Published:** 2026-11-06

**Authors:** Ying Shi, Han Zhang, Yuhang Jiang, Xin Li, Daqiang Sun

**Affiliations:** 1 Chest Hospital, Tianjin University, Tianjin 300072, China; shiyingdd@126.com (Y.S.); pumpkin2018@tmu.edu.cn (H.Z.); yhjiang2023@163.com (Y.J.); 2 Tianjin Institute of Cardiovascular Disease, Tianjin Chest Hospital, Tianjin 300222, China; 3 Department of Thoracic Surgery, Tianjin Chest Hospital, Tianjin 300222, China

**Keywords:** lung adenocarcinoma, ANGPTL4, cellular senescence, macrophages, tumor microenvironment, NLRP3 signaling

## Abstract

**Background**: Cellular senescence and the senescence-associated secretory phenotype (SASP) can alter communication between tumor cells and the surrounding microenvironment, but the extracellular mediators involved in this process remain poorly defined in lung adenocarcinoma (LUAD). **Methods**: Bulk transcriptomic analysis was used to characterize senescence-related transcriptional alterations and construct a prognostic signature. ANGPTL4 expression was evaluated in public datasets and clinical samples. Single-cell RNA-sequencing analysis was then performed to map senescence-associated gene-set scores across LUAD cell populations and to examine the cell–cell communication. ANGPTL4 depletion and overexpression were used to assess its effects on LUAD-cell proliferation, migration, and SA-β-gal positivity, while a subcutaneous xenograft model was used to examine tumor growth in vivo. Conditioned-medium and indirect co-culture experiments were performed to assess the effects of ANGPTL4-overexpressing LUAD cells on THP-1-derived macrophage inflammatory responses, and MCC950 was used to evaluate the contribution of NLRP3-associated signaling. **Results**: Senescence-related transcriptional alterations in LUAD were enriched in inflammatory and immune-related pathways. ANGPTL4 was included in a five-gene prognostic model and selected for functional investigation because it encodes a secreted protein. We found that ANGPTL4 protein levels were increased in LUAD tissues. Single-cell analysis showed relatively high senescence-associated gene-set scores in macrophage-enriched populations, together with stronger cell–cell communication in the higher-score group. ANGPTL4 depletion reduced LUAD-cell migration and SA-β-gal positivity, while its overexpression had the opposite effects and resulted in larger xenograft tumors. Conditioned medium and indirect co-culture experiments showed that ANGPTL4-overexpressing LUAD cells induced inflammatory changes in THP-1-derived macrophages, accompanied by increased NLRP3-associated markers and IL-1β secretion. These changes were partly attenuated by MCC950. **Conclusions**: These findings suggest that ANGPTL4 is associated with tumor cell–macrophage crosstalk in LUAD and may correlate with senescence-related transcriptional signatures, while the specific biological role of ANGPTL4 remains to be further characterized.

## Simple Summary

Lung adenocarcinoma develops through interactions between cancer cells and surrounding immune cells. We investigated ANGPTL4, a secreted protein identified through analyses of genes associated with cellular senescence, a stress response often linked to persistent growth arrest. Using public gene expression datasets, patient samples, and experimental models, we found increased ANGPTL4 levels in lung adenocarcinoma. Altering ANGPTL4 expression changed cancer cell growth, migration, and SA-β-gal positivity, although these findings did not establish a complete senescence program. Increasing ANGPTL4 expression also enhanced tumor growth in mice. Culture medium collected from these cancer cells altered inflammatory markers in a laboratory macrophage model. Some changes were reduced by an NLRP3 inhibitor. These findings support further investigation of ANGPTL4-related tumor-immune interactions, while additional studies are needed to determine whether ANGPTL4 itself causes the immune-cell responses.

## 1. Introduction

Lung cancer remains one of the leading causes of cancer-related mortality worldwide, and lung adenocarcinoma (LUAD) is the most common histological subtype of non-small-cell lung cancer (NSCLC) [1,2]. Despite advances in targeted therapies and immune checkpoint inhibitors, therapeutic resistance and disease progression remain major challenges in LUAD [1,3]. Interactions between malignant cells and surrounding stromal and immune populations also contribute to the biology of LUAD [4]. Tumor-associated macrophages are abundant and highly plastic components of the lung cancer microenvironment and can influence tumor growth, invasion, angiogenesis, and antitumor immunity [5,6,7]. Identifying tumor-derived factors that influence macrophage inflammatory responses may help clarify their interactions with LUAD cells.

Cellular senescence is relevant to these interactions because it can alter both cell proliferation and the secretion of factors that act on neighboring cells [8,9]. Cellular senescence is characterized by durable cell-cycle arrest, but senescent cells can remain metabolically active and develop a senescence-associated secretory phenotype (SASP) [10,11,12]. SASP factors include cytokines, chemokines, growth factors, and matrix-remodeling molecules that affect neighboring cells [13]. Single-cell studies have revealed diverse myeloid populations in lung tumors, highlighting the need to examine inflammatory features within specific cellular compartments [14]. These observations motivate the search for secreted factors associated with senescence-related features and macrophage responses in LUAD.

Angiopoietin-like 4 (ANGPTL4) is a secreted glycoprotein involved in lipid metabolism, vascular permeability, and tissue remodeling [15]. Beyond its metabolic functions, ANGPTL4 has been implicated in tumor cell survival, vascular leakage, and invasion [16,17,18]. Emerging studies have also linked ANGPTL4 to NSCLC progression, metabolic adaptation, metastasis, and therapeutic resistance [19,20,21]. Its extracellular localization and potential involvement in stress- and aging-related processes [22] raise the possibility that ANGPTL4 may contribute to paracrine communication within the LUAD microenvironment. Nevertheless, the relationship between ANGPTL4 and senescence-associated states in LUAD has not been clearly established. Whether ANGPTL4-overexpressing LUAD cells alter macrophage inflammatory responses remains unclear.

Here, we combined bulk and single-cell transcriptomic analyses with clinical sample assessment and functional experiments. A senescence-related prognostic model was used to prioritize ANGPTL4 for further study. We examined the effects of ANGPTL4 depletion and overexpression on LUAD-cell proliferation, migration, and SA-β-gal positivity, and assessed xenograft growth following ANGPTL4 overexpression. Conditioned-medium and indirect co-culture experiments were used to test macrophage responses to ANGPTL4-overexpressing LUAD cells, with MCC950 used to examine the contribution of NLRP3 signaling.

## 2. Materials and Methods

### 2.1. Data Acquisition

Senescence-related gene sets (SenMayo) were retrieved from a published study [23]. Bulk RNA-seq data for LUAD were sourced from TCGA-LUAD and six GEO cohorts (GSE13213, GSE26939, GSE30219, GSE31210, GSE42127, GSE50081). Non-tumor tissues and samples with incomplete survival information were excluded. Immunotherapy-related indicators were collected from the Immunophenoscore (IPS; The Cancer Immunome Atlas, TCIA; https://tcia.at/, accessed on 11 May 2025) and the Tumor Immune Dysfunction and Exclusion (TIDE; http://tide.dfci.harvard.edu/, accessed on 11 May 2025) scores. Single-cell RNA-sequencing data were downloaded from the Genome Sequence Archive for Human (GSA-Human; accession HRA002509). Batch effects across bulk RNA-seq datasets were corrected using the *sva (v3.50.0)* package, and Harmony (v1.1.0) was applied to remove inter-sample variation in single-cell transcriptomes.

### 2.2. Construction and Validation of the Prognostic Model

Senescence-related genes were differentially analyzed between tumor and normal tissues in TCGA-LUAD. Differentially expressed genes were subjected to univariate Cox regression, followed by LASSO-Cox regression to build a multigene prognostic signature. Risk scores were calculated for each patient, and patients were stratified into high- and low-risk groups by the median score. Kaplan–Meier analysis and ROC curves assessed survival differences and predictive accuracy. Model validation was performed across multiple GEO cohorts.

### 2.3. Enrichment Analyses

Gene set variation analysis (GSVA) was performed using HALLMARK gene sets from the Molecular Signatures Database (MSigDB). Gene set enrichment analysis (GSEA) was conducted using GO and KEGG gene sets. All enrichment analyses were implemented via the *clusterProfiler (v4.10.0)* and *GSVA (v1.50.0) packages*.

### 2.4. Immunotherapy Response Prediction

IPS and TIDE scores were compared between high- and low-risk groups. IPS reflects tumor immunogenicity associated with enhanced sensitivity to immune checkpoint inhibitors (ICIs). TIDE integrates T-cell dysfunction and exclusion signatures, with higher scores indicating poor ICI response. Differences in immune checkpoint molecules, DNA methylation, and copy number variation (CNV) were also assessed.

### 2.5. Drug Sensitivity Analysis

Drug response prediction was conducted using drug sensitivity data from the Genomics of Drug Sensitivity in Cancer (GDSC) database. The *pRRophetic (v0.50)* package was employed to estimate the half-maximal inhibitory concentration (IC50) for various chemotherapeutic and targeted agents by modeling the relationship between gene expression and drug sensitivity in cancer cell lines. Predicted IC50 values were compared between the two risk groups.

### 2.6. Tumor Microenvironment Evaluation

TME was characterized using seven established deconvolution algorithms, including TIMER, CIBERSORT, CIBERSORT-ABS, QUANTISEQ, MCPCOUNTER, XCELL, and EPIC. The ESTIMATE algorithm was further applied to compute ImmuneScore, StromalScore, and ESTIMATEScore, from which tumor purity was inferred.

### 2.7. Single-Cell RNA-Seq Processing

Raw scRNA-seq data were processed using Seurat (v4.4.0). A Seurat object was constructed, followed by normalization and identification of highly variable genes. Low-quality cells were filtered based on gene/UMI counts and mitochondrial/hemoglobin gene expression. After normalization and scaling, cell cycle scoring was performed to minimize cell cycle-related biases. Harmony (v1.1.0) was applied for batch correction. PCA, graph-based clustering, and UMAP visualization were performed. Senescence-associated transcriptional scores were calculated using AUCell (v1.24.0), UCell (v2.6.2), singscore (v1.22.0), AddModuleScore, and single-sample gene set enrichment analysis (ssGSEA, implemented in GSVA (v1.50.0)). These gene-set scores were interpreted as transcriptional features rather than direct evidence of cellular senescence.

Epithelial-cell copy-number profiles were inferred jointly across samples using inferCNV (v1.18.0), with T cells as the reference population and hg19 gene coordinates. Raw RNA counts were analyzed with cutoff = 0.1, cluster_by_groups = TRUE, denoise = TRUE, HMM = FALSE, and min_cells_per_gene = 10. K-means clustering (k = 5) was applied to the resulting CNV profiles. Clusters 2–5 were classified as putatively malignant based on their coordinated chromosomal alterations and elevated CNV scores relative to T cells. The remaining epithelial cells were operationally classified as putatively nonmalignant; this designation was not independently validated.

For patient-level analysis, postoperative samples containing at least 30 putatively malignant epithelial cells and 30 macrophages were retained. ANGPTL4 expression was averaged over malignant cells, and NLRP3, IL1B, CASP1, and GSDMD expression was separately averaged over macrophages from the same patient, using log-normalized RNA expression and retaining zero-expression cells. Six patients met these criteria. Each patient contributed one paired expression summary to the correlation analysis.

### 2.8. Cell–Cell Communication Analysis

Cells were stratified into high- and low-senescence activity groups by median single-cell senescence score. CellChat (v2.2.0.9001) inferred ligand–receptor networks separately for each group. For each group, the number of ligand–receptor pairs, signaling pathway activity, and contributions of specific sender and receiver cell types were quantified. Comparisons were made in terms of signaling strength, pathway activity, and sender/receiver contributions.

### 2.9. Cell Culture

The cell lines used in this study included A549 (RRID:CVCL_0023), NCI-H1299 (RRID:CVCL_0060), NCI-H1650 (RRID:CVCL_1483), NCI-H1975 (RRID:CVCL_1511), BEAS-2B (RRID:CVCL_0168), and THP-1 (RRID:CVCL_0006). Cells were cultured in RPMI-1640 medium (Gibco, Waltham, MA, USA) supplemented with 10% fetal bovine serum (ExCell, Suzhou, China) and 1% penicillin/streptomycin (Yeason, Shanghai, China). All cells were incubated in a humidified atmosphere containing 5% CO_2_ at 37 °C. All cell lines were recently authenticated by STR profiling and confirmed to be mycoplasma-free.

### 2.10. siRNA Transfection and Lentiviral Transduction

For gene knocking down, small interfering RNAs (si-ANGPTL4 or si-NC) were synthesized by (Sangon, Shanghai, China). Cells were seeded in 6-well plates and cultured until 50–60% confluence. Transient transfection was performed using Lipofectamine 3000 reagent (Invitrogen, Carlsbad, CA, USA) according to the manufacturer’s instructions. To establish cell lines with stable overexpression of ANGPTL4, lentiviral particles (LV-ANGPTL4 or LV-GFP, BrainVTA, Wuhan, China) were used, followed by puromycin selection for ≥2 weeks. Knockdown and overexpression efficiencies were confirmed by RT-qPCR and Western blotting. The sequences of si-*ANGPTL4* are as follows: #1: gcgaauucagcaucugcaa (sense), uugcagaugcugaauucgc (antisense); #3: cguccugggacagaugaa (sense), uucaucucgucccaggacg (antisense).

### 2.11. RT-qPCR and Western Blotting

Total RNA was extracted and reverse-transcribed using commercial reagents according to the manufacturer’s instructions (Yeason, Shanghai, China). RT-qPCR was performed using SYBR Green Master Mix (Yeason, Shanghai, China) on an ABI 7500 Real-Time PCR System. Target gene expression was normalized to GAPDH using the 2^−ΔΔCt^ method. The sequences of primers are shown as follows:

human *ANGPTL4*: Forward, ATGGAGGCTGGACAGTAATTCAG; Reverse, GCTATGCACCTTCTCCAGACC; human *CD86*: Forward, CTGCTCATCTATACACGGTTACC; Reverse, GGAAACGTCGTACAGTTCTGTG; human *CD206*: Forward, GTGATGGGACCCCTGTAACG; Reverse, CTGCCCAGTACCCATCCTTG; human *CCL2*: Forward, CCCCAGTCACCTGCTGTTAT; Reverse, CTTCTTTGGGACACTTGCTGC; human *PD-L1*: Forward, CATGTCAGGCTGAGGGCTAC; Reverse, TGGAATTGGTGGTGGTGGTC; human *GAPDH*: Forward, GGAAGCTTGTCATCAATGGAAATC; Reverse, TGATGACCCTTTTGGCTCCC.

Total protein was extracted using RIPA lysis buffer supplemented with protease and phosphatase inhibitors. Protein concentrations were determined using a BCA Protein Assay Kit (Solarbio, Beijing, China). Equal amounts of protein (30 μg) were separated by SDS-PAGE and transferred to PVDF membranes (Millipore, Burlington, MA, USA). Membranes were blocked with 5% non-fat milk and incubated overnight at 4 °C with primary antibodies: GAPDH, α-tubulin (1:10,000, Proteintech, Wuhan, China), ANGPTL4, NLRP3, GSDMD, IL1β, CD86, E-cad, Slug, Twist (1:1000, Proteintech, Wuhan, China). After incubation with HRP-conjugated secondary antibodies, protein bands were visualized using ECL substrate (Yeason, Shanghai, China). The original uncropped WB images are provided in the File S1 of Supplementary Materials.

### 2.12. CCK-8 Assay

Cells were seeded in 96-well plates (1000 cells/well) and incubated for 24, 48, 72, and 96 h. At each time point, 10 μL of CCK-8 reagent (Yeason, Shanghai, China) was added to each well, followed by incubation for 1 h. Absorbance was measured at 450 nm using a microplate reader.

### 2.13. Colony Formation Assay

Lung cancer cells were seeded in 6-well plates (1000 cells/well) and cultured for 10–14 days. Colonies were fixed with 4% paraformaldehyde and stained with 0.1% crystal violet.

### 2.14. Wound Healing Assay

Lung cancer cells were grown to 90% confluence in 6-well plates. A longitudinal scratch was made using a 200-μL pipette tip. Migration distance was monitored at 0 and 24 h using an inverted microscope.

### 2.15. Transwell Migration Assay

Serum-free cells (5 × 10^4^) were added to upper transwell chambers (8-μm pores), while medium containing 10% FBS was placed in the lower chamber. After 24 h, migrated cells on the lower surface were fixed, stained with crystal violet, and quantified in five random fields.

### 2.16. Senescence-Associated β-Galactosidase (SA-β-Gal) Staining

SA-β-gal activity was detected using an SA-β-gal Staining Kit (Beyotime, Shanghai, China) according to the manufacturer’s instructions. Briefly, cells were fixed for 15 min at room temperature and incubated overnight at 37 °C (without CO_2_) with the SA-β-gal staining solution at pH 6.0. The proportion of SA-β-gal-positive cells was quantified under a light microscope.

### 2.17. EdU Proliferation Assay

Cells were incubated with 10 µM EdU for 2–4 h, then fixed, permeabilized, and stained using a click chemistry reaction. Nuclei were counterstained with Hoechst 33342. EdU-positive cells were counted from five random fields per well.

### 2.18. Conditioned Medium (CM) Collection

Stably transfected cells were cultured to 80% confluence, washed three times with PBS, and incubated in serum-free medium for 24 h. The supernatant was collected, centrifuged at 3000 rpm for 10 min to remove cell debris, and filtered through a 0.22 μm PVDF membrane. The CM was stored at −80 °C for subsequent experiments. Undiluted CM was used for direct stimulation of THP-1 macrophages and the MCC950 inhibition assay. For A549 migration assays, both undiluted CM and 1:1 diluted CM were applied.

### 2.19. Macrophage Differentiation and Co-Culture Models

Human THP-1 monocytes were differentiated into M0 macrophages by incubation with 100 nM PMA (Sigma-Aldrich, St. Louis, MO, USA) for 24 h. Then cells were washed three times with PBS and cultured in fresh RPMI-1640 medium for an additional 24 h to reach a quiescent state. For co-culture, ANGPTL4-overexpressing or control LUAD cells (2 × 10^5^) were seeded in upper transwell inserts (0.4 μm), placed over THP-1 macrophages (1 × 10^6^) in lower chambers, and co-cultured for 48 h. For CM treatment experiments, PMA-differentiated THP-1 macrophages were directly cultured in undiluted lung cancer-derived CM for 48 h.

### 2.20. NLRP3 Inhibition with MCC950

Differentiated THP-1 macrophages were pre-treated with 10 μM MCC950 (MCE, Shanghai, China) for 2 h. Subsequently, the cells were exposed to undiluted CM derived from ANGPTL4-overexpressing lung cancer cells for another 48 h.

### 2.21. Subcutaneous Xenograft Tumor Model

All animal procedures were approved by the Institutional Animal Care and Use Committee of Tianjin Chest Hospital (TJCH-2024-017) and conformed to the Guide for the Care and Use of Laboratory Animals of the US National Institutes of Health (NIH Publication No. 85–23, updated 2011). Male BALB/c nude mice (4–6 weeks old) were injected subcutaneously with A549 cells stably overexpressing ANGPTL4 or empty vector (1 × 10^7^ cells mixed with 100 μL Matrigel). Tumor dimensions were measured every 3–4 days. Mice were euthanized at 4 weeks. Tumors were excised, weighed, photographed, and processed for further molecular analyses.

### 2.22. Human Specimens

Human LUAD paired adjacent non-tumor tissues and serum samples were obtained from Tianjin Chest Hospital. The study was approved by the Medical Ethics Committee of Tianjin Chest Hospital (approval number: 2026ys-059).

### 2.23. Immunohistochemistry (IHC)

Paraffin-embedded tissue sections were deparaffinized, rehydrated, and subjected to antigen retrieval. After blocking endogenous peroxidase activity and nonspecific binding, sections were incubated with ANGPTL4 antibodies (1:100, Proteintech, China) and corresponding secondary detection reagents, followed by DAB visualization and hematoxylin counterstaining. DAB-positive area was quantified as a percentage of the total tissue area.

### 2.24. Enzyme-Linked Immunosorbent Assay (ELISA)

ANGPTL4 and IL1β concentrations in condition medium or human plasma were measured using a commercial ELISA kit (MultiSciences, Hangzhou, China) according to the manufacturer’s instructions. Absorbance was measured at 450 nm using a microplate reader.

### 2.25. Statistical Analysis

Computational analyses were performed in R (v4.3.1; additional inferCNV analysis, v4.5.3). Differential gene expression was assessed using the *limma* (*v3.58.1*) package, with false discovery rate (FDR) < 0.05 considered significant. Survival outcomes were evaluated using Kaplan–Meier curves with log-rank tests. Univariate and multivariate Cox regression analyses were used to estimate hazard ratios and 95% confidence intervals. Model development and evaluation employed LASSO regression, ROC curve analysis, and calculation of the concordance index (C-index) using the *survminer* (*v0.4.9*) and *timeROC* (*v0.4*) packages. Immune infiltration analyses incorporated multiple deconvolution methods and the ESTIMATE algorithm.

For the analysis of senescence-associated gene-set scores, the statistical unit was the individual cell, using an overall Kruskal–Wallis test and pairwise two-sided Wilcoxon rank-sum tests without multiplicity adjustment. These descriptive comparisons pooled available time points and did not account for within-patient dependence. Malignant versus nonmalignant epithelial ANGPTL4 expression was compared using a two-sided Wilcoxon rank-sum test. Comparisons of the six epithelial score metrics used Wilcoxon tests with Benjamini–Hochberg adjustment. Patient-level expression associations were evaluated using Spearman correlation, with Benjamini–Hochberg adjustment across the four macrophage genes.

In vitro data are presented as mean ± SEM from at least three independent experiments. Technical replicate measurements and multiple image fields were not counted as independent biological replicates. Statistical analyses were performed using GraphPad Prism 9.0. Differences between two groups were analyzed using Student’s *t*-test, while comparisons among multiple groups were performed using one-way ANOVA followed by Tukey’s multiple comparisons test. *p* < 0.05 was considered statistically significant.

## 3. Results

### 3.1. Senescence-Associated Transcriptional Features in LUAD and ANGPTL4 Emerged as a Candidate Mediator

**Figure 1:**
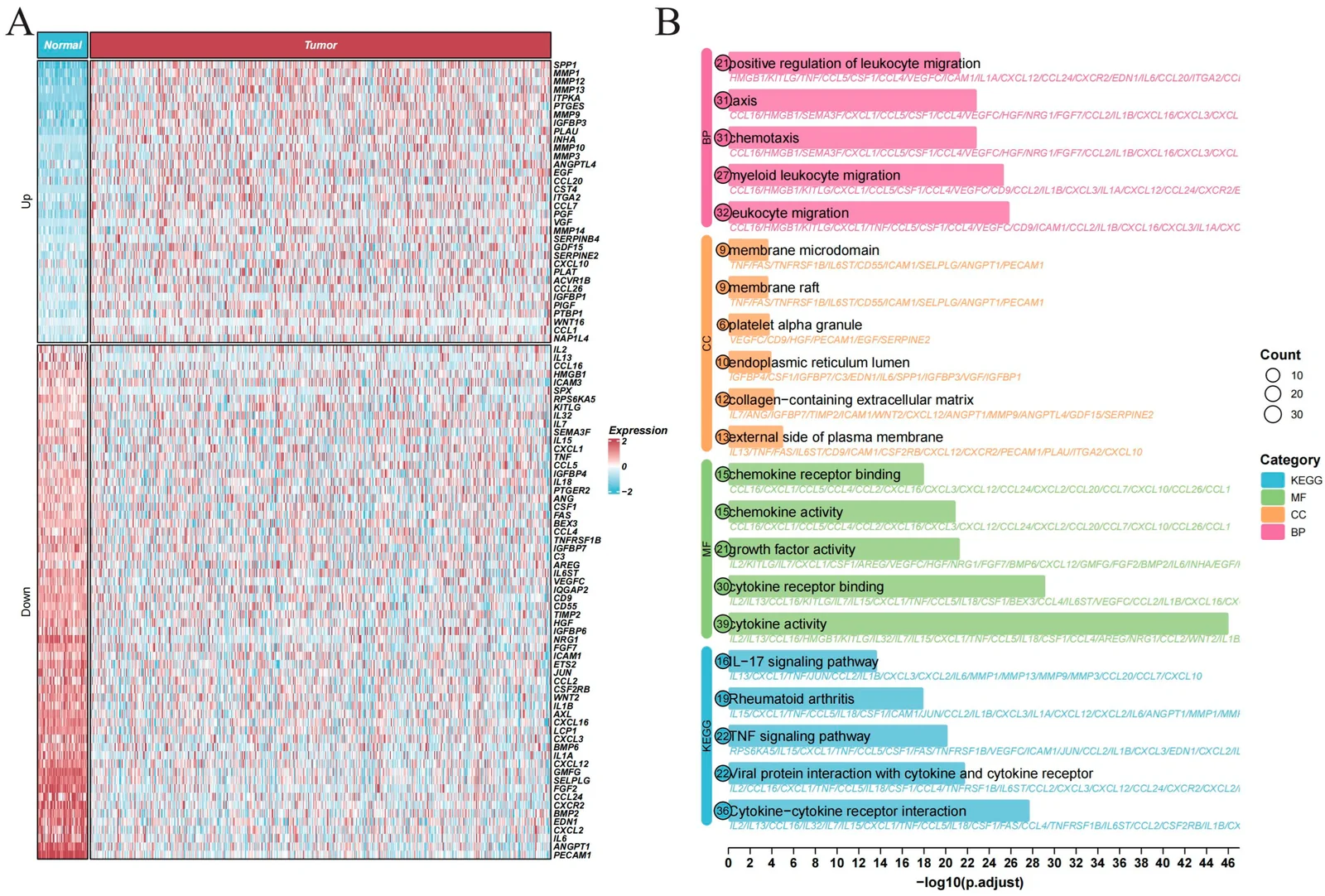
Differential expression landscape and functional annotation of senescence-related genes in TCGA-LUAD. (**A**) Heatmap of differentially expressed senescence-related genes in TCGA-LUAD cohort (*p* < 0.05) in normal vs. tumor tissues. Genes are organized into upregulated and downregulated categories. (**B**) Functional annotation of the differentially expressed genes, including Gene Ontology biological process, cellular component, molecular function terms, and KEGG pathways. Dot size indicates gene count; *x*-axis shows –log10 (p.adjust).

To characterize senescence-associated transcriptional features in lung adenocarcinoma (LUAD), we first compared the expression profiles of senescence-related genes between tumor and normal tissues. Hierarchical clustering revealed distinct expression patterns between the two groups, with genes such as SPP1 and MMP1 showing higher expression in tumor tissues (Figure 1A). Functional enrichment analysis indicated that the differentially expressed genes were associated with leukocyte migration, cytokine activity, and chemokine receptor binding. KEGG analysis further identified inflammatory pathways, including the IL-17 signaling pathway and cytokine–cytokine receptor interaction (Figure 1B). These findings suggest that senescence-associated transcriptional alterations in LUAD are accompanied by inflammatory and microenvironment-related gene programs.

**Figure 2:**
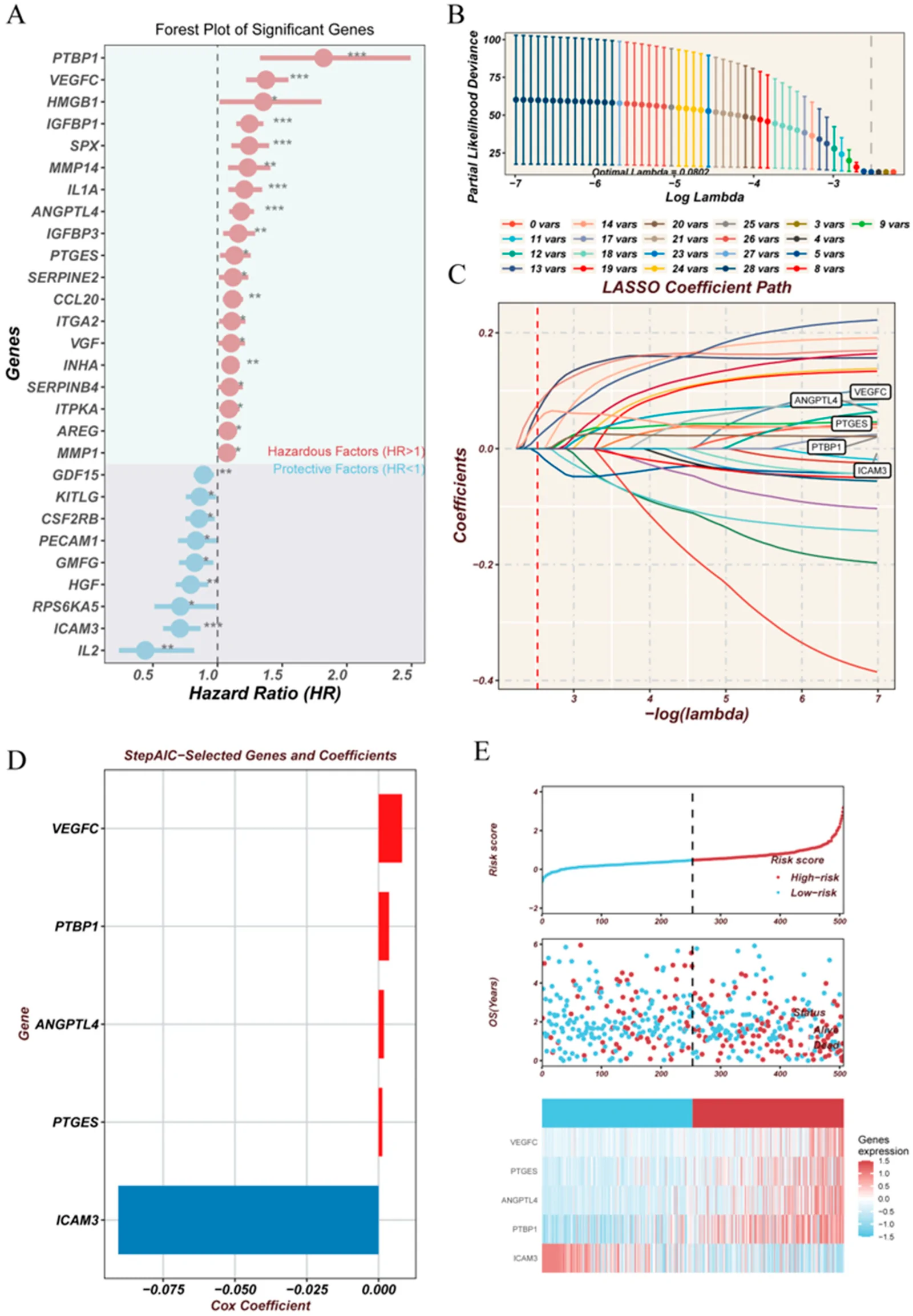
Prognostic gene selection, model construction, and risk characteristics. (**A**) Univariate Cox regression of differentially expressed genes in TCGA-LUAD. (**B**) LASSO cross-validation plot showing partial likelihood deviance across λ values. (**C**) LASSO coefficient trajectory plot, displaying the variation in gene coefficients under different λ parameters. (**D**) Genes included in the multivariate Cox regression model and their corresponding risk coefficients. (**E**) Risk score distribution, patient survival status, and expression profiles of selected genes. * *p* < 0.05, ** *p* < 0.01, *** *p* < 0.001.

We next evaluated the prognostic relevance of the differentially expressed senescence-related genes in the TCGA-LUAD cohort. Univariate Cox regression identified several genes associated with overall survival, including PTBP1, VEGFC, HMGB1, and IGFBP1, whereas ICAM3 and IL2 were associated with lower estimated risk (Figure 2A). LASSO-Cox regression was subsequently used to reduce the number of candidate genes, with the optimal penalty parameter selected by cross-validation (Figure 2B,C). A five-gene prognostic model was constructed using multivariable Cox regression and included VEGFC, PTBP1, ANGPTL4, PTGES, and ICAM3 (Figure 2D). VEGFC, PTBP1, ANGPTL4, and PTGES had positive coefficients, whereas ICAM3 had a negative coefficient. In the TCGA cohort, patients with higher risk scores showed poorer overall survival and a higher frequency of death events (Figure 2E). Among the genes retained in the prognostic model, ANGPTL4 was selected for further study because it encodes a secreted protein with a potential role in communication between tumor cells and the microenvironment.

The prognostic model was further evaluated in six independent GEO cohorts, including GSE13213, GSE26939, GSE30219, GSE50081, GSE31210 and GSE42127. Time-dependent ROC discrimination varied across cohorts and prediction horizons, with 1-, 3-, and 5-year AUCs ranging from 0.560 to 0.791 (Supplementary Figure S1). Performance was weaker in GSE31210, precluding a claim of uniformly stable discrimination. The high-risk group was enriched for cell-cycle and proliferative programs, including G2M checkpoint, E2F targets, MYC signaling, and spliceosome-related pathways (Supplementary Figure S2). The two risk groups also differed in immune checkpoint expression, antigen-presentation-related genes, predicted drug-response profiles, IPS, and TIDE scores. The low-risk group showed higher IPS values, whereas the high-risk group showed higher TIDE scores (Supplementary Figure S3). Immune deconvolution analyses showed generally higher immune-cell infiltration in the low-risk group, whereas high-risk tumors had lower ImmuneScore and StromalScore and higher estimated tumor purity (Supplementary Figure S4). In the additional TCGA-LUAD Cox analysis (489 patients; 178 events), the risk score was associated with overall survival in the univariate model (HR per standard-deviation increase, 1.67; 95% CI, 1.47–1.90; *p* < 0.001). The association persisted after adjustment for age, sex, and stage (adjusted HR, 1.62; 95% CI, 1.41–1.86; *p* < 0.001; Supplementary Figure S5).

### 3.2. ANGPTL4 Protein Levels Are Elevated in LUAD Tissues and Patient Serum

**Figure 3:**
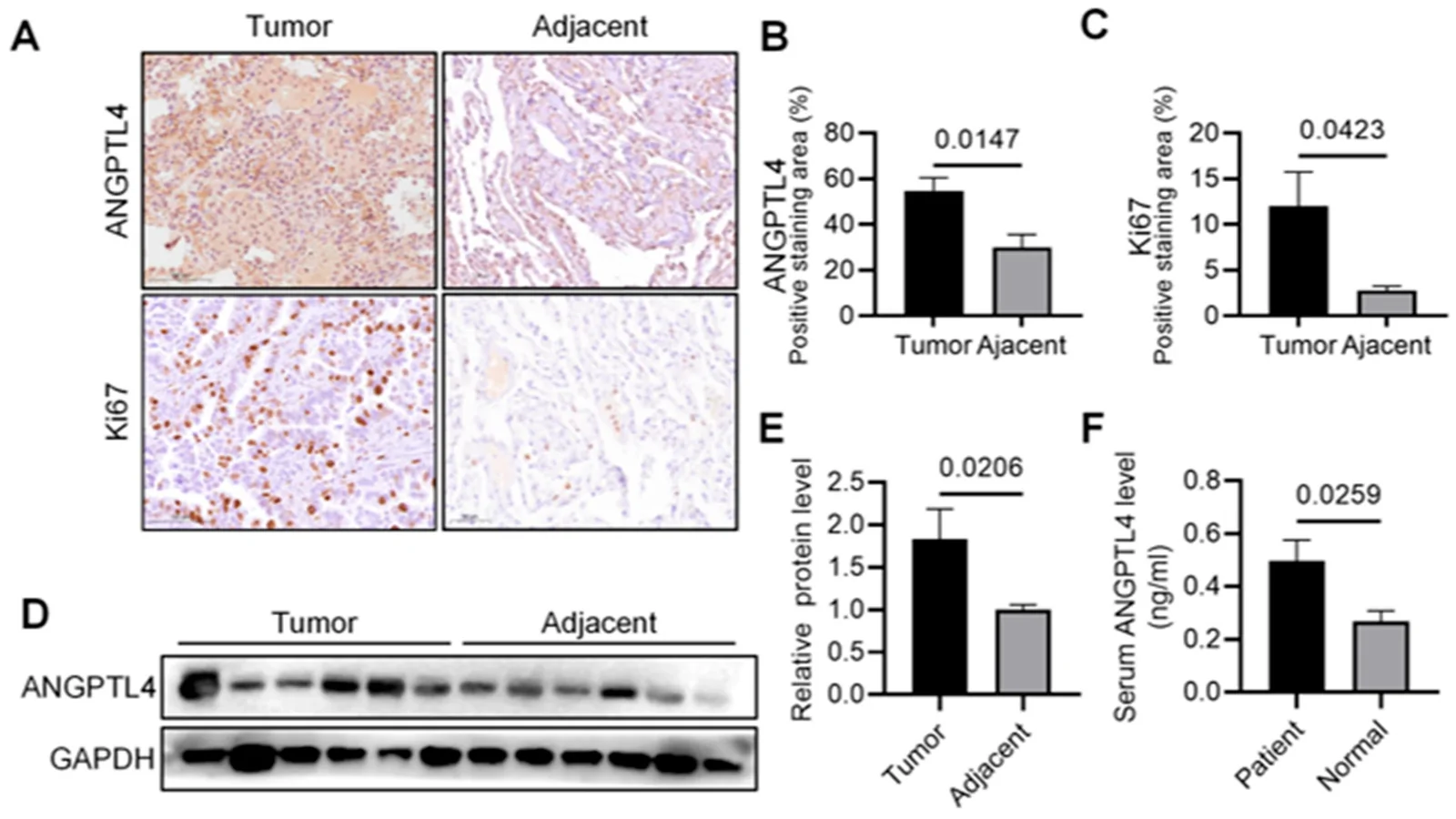
ANGPTL4 protein levels are elevated in LUAD tissues and patient serum (**A**–**C**) Representative IHC images and qualification of ANGPTL4 and Ki-67 in LUAD vs. adjacent normal tissues (LUAD tissues: *n* = 9, adjacent tissues: *n* = 5), scale bar, 50 μm. (**D**,**E**) Western blot and quantification of ANGPTL4 in paired LUAD and adjacent normal samples (*n* = 6). (**F**) ELISA of serum ANGPTL4 level of LUAD patients vs. controls (*n* = 9), the controls for the ELISA were patients with benign pulmonary conditions. Data are presented as mean ± SEM.

We next examined ANGPTL4 protein levels in clinical LUAD samples. Immunohistochemical analysis revealed stronger ANGPTL4 staining in LUAD tissues than in adjacent non-tumor tissues. Ki-67 staining was also higher in tumor tissues (Figure 3A–C). Western blot analysis of paired tumor and adjacent non-tumor samples further supported increased ANGPTL4 protein abundance in LUAD tissues (Figure 3D,E). Serum ELISA showed higher circulating ANGPTL4 concentrations in patients with LUAD than in control individuals (Figure 3F). Together, these observations provide evidence of increased ANGPTL4 protein abundance in the examined LUAD tissues and elevated circulating ANGPTL4 levels in the patient cohort, supporting further investigation of its potential role in LUAD.

### 3.3. Single-Cell Analysis Reveals Higher Senescence-Associated Gene-Set Scores in Macrophage and Monocyte Populations

**Figure 4:**
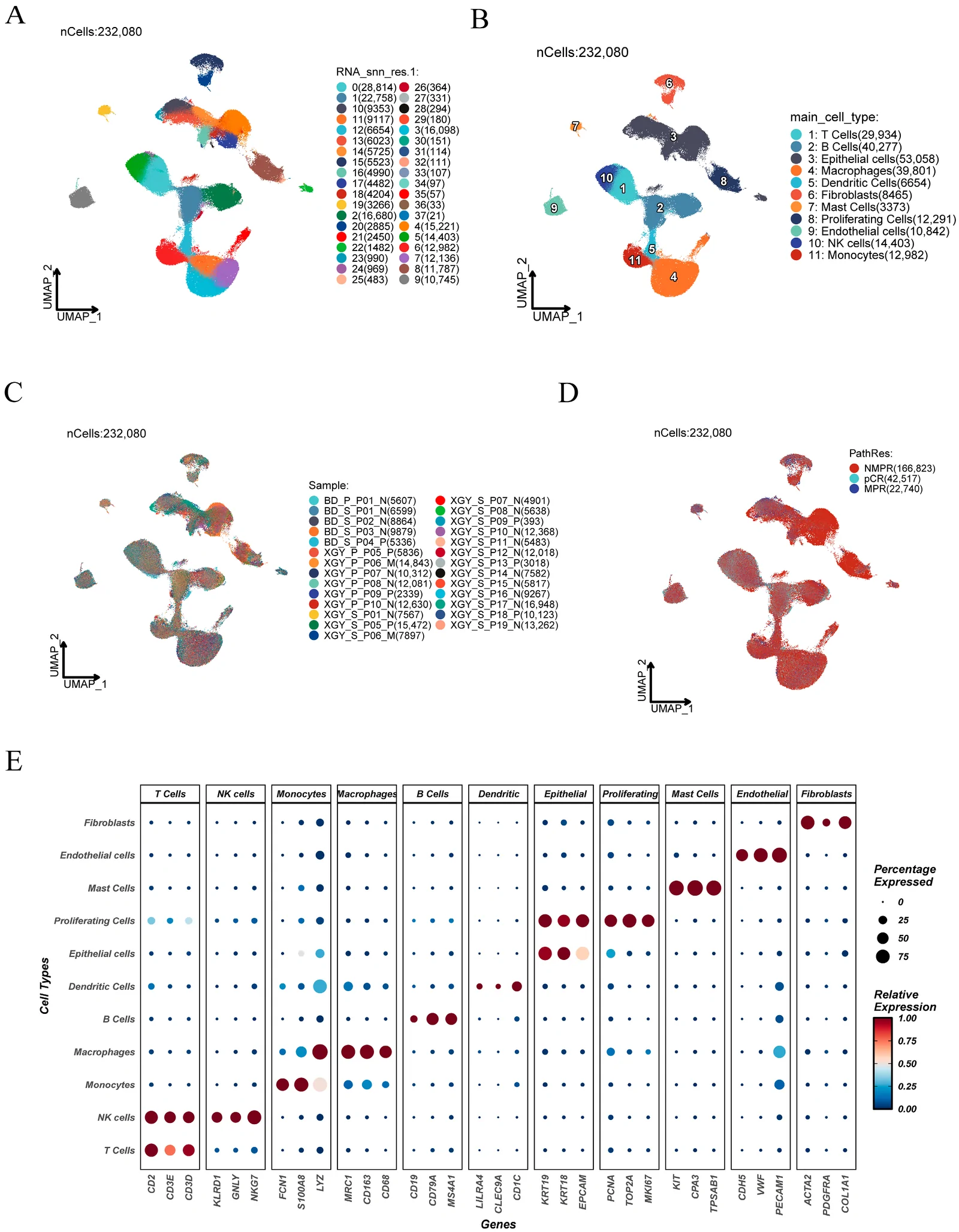
Cell populations and sample distribution in the single-cell RNA-sequencing dataset. (**A**) UMAP of single-cell transcriptomes showing distinct clusters. (**B**) Cell-type annotation based on canonical marker genes. (**C**) UMAP visualization of cells grouped by sample origin. (**D**) Distribution of cells across different immunotherapy response groups. (**E**) Bubble plot showing expression patterns of canonical marker genes across major cell types, including expression proportion and relative expression level.

To examine the cellular distribution of senescence-associated features in the tumor microenvironment, we analyzed the NSCLC single-cell dataset HRA002509 [24]. After quality control and batch correction (Supplementary Figure S6), dimensionality reduction and clustering identified eleven major cell populations, including T cells, NK cells, monocytes, macrophages, B cells, dendritic cells, epithelial cells, proliferating cells, mast cells, endothelial cells, and fibroblasts (Figure 4A,B). Cell-type assignments were supported by canonical markers, including CD3D/CD3E for T cells, MS4A1 for B cells, LYZ/CD68 for macrophages, EPCAM for epithelial cells, and COL1A1/COL1A2 for fibroblasts (Figure 4C–E).

**Figure 5:**
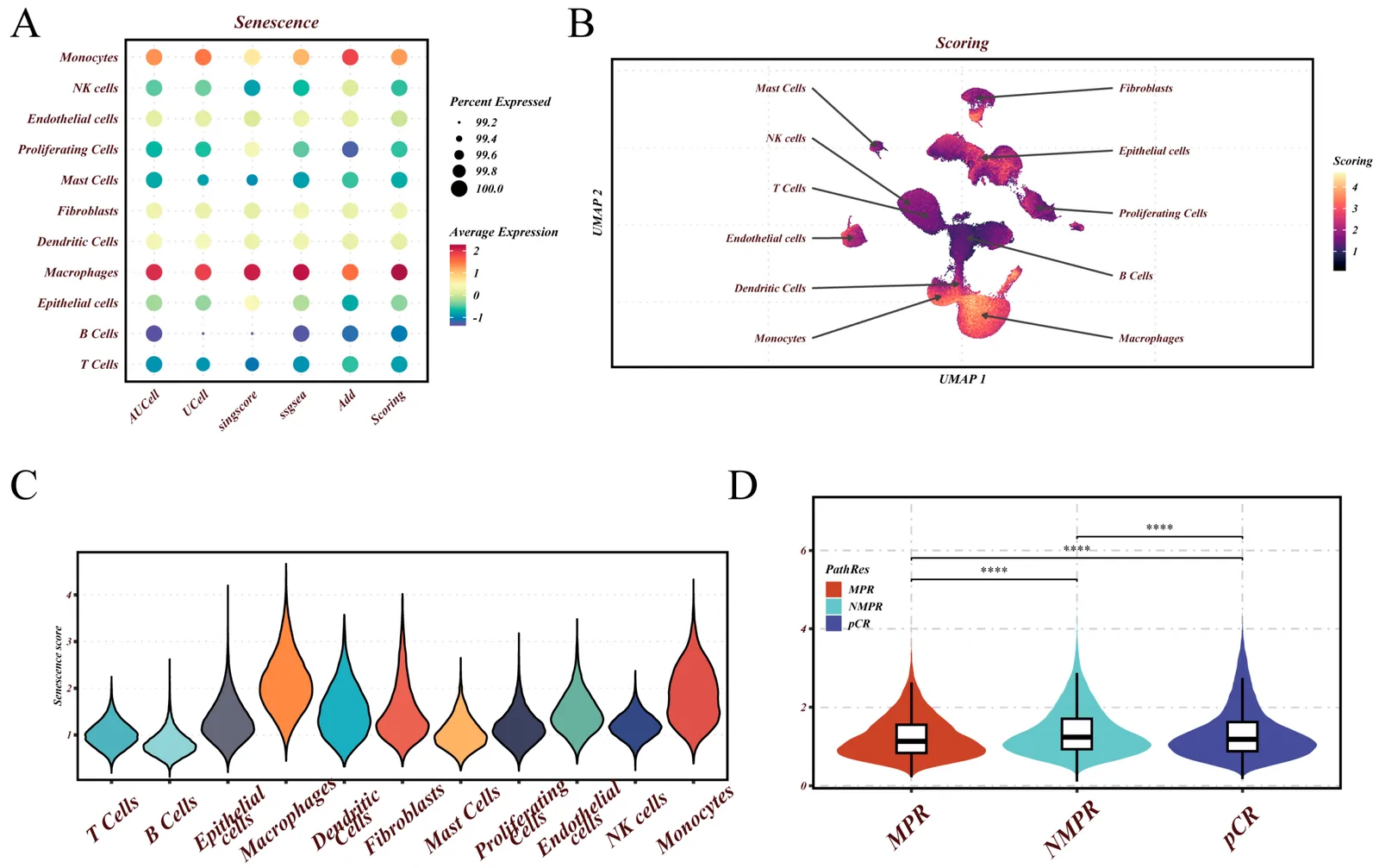
Distribution of senescence-associated gene-set scores across cell populations and pathological response groups. (**A**) Dot plot of five scoring methods (AUCell, UCell, singscore, ssGSEA, and AddModuleScore) and the composite score (Scoring). (**B**) UMAP visualization colored by the composite score, with repositioned cell-type labels and arrows identifying the corresponding cell groups. (**C**) Cell-level score distributions across the eleven annotated cell types. (**D**) Cell-level composite-score distributions by pathological response (MPR, pCR, and NMPR), pooling available pretreatment and postoperative samples. Each observation is a cell, not an independent patient. The overall comparison used a Kruskal–Wallis test; pairwise comparisons used two-sided Wilcoxon rank-sum tests without multiplicity adjustment. Boxes indicate medians and interquartile ranges. Significance labels denote **** *p* < 0.0001. The response groups included 1 MPR, 5 pCR, and 13 NMPR patients.

Senescence-associated gene-set scores were then estimated using five complementary scoring approaches: AUCell, UCell, singscore, AddModuleScore, and GSVA. Across the five scoring methods, macrophages and monocytes generally had higher scores than B- and T-cell populations (Figure 5A). UMAP and violin-plot analyses further demonstrated that higher scores were concentrated in macrophage- and monocyte-enriched regions (Figure 5B,C). At the individual-cell level, cells from NMPR cases showed higher composite scores than those from MPR and pCR cases (Figure 5D). These pooled cell-level comparisons are descriptive and do not establish patient-level differences in treatment response, particularly because the MPR category contained only one patient.

CellChat analysis suggested stronger inferred ligand–receptor communication in the higher-score group than in the lower-score group, particularly among macrophages, monocytes, fibroblasts, and epithelial cells (Supplementary Figure S7). Overall, the single-cell data associated higher senescence-related scores with myeloid-enriched compartments, motivating further exploratory analysis of epithelial cell–macrophage associations.

Joint inferCNV analysis identified epithelial subgroups with elevated and coordinated CNV signals relative to the T-cell reference (Supplementary Figure S8A–C). Based on these patterns, epithelial cells in k-means clusters 2–5 were classified as putatively malignant (21,850 cells), while the remaining 31,208 epithelial cells were treated as putatively nonmalignant. Senescence-associated score differences between these groups were method-dependent rather than uniformly elevated in malignant cells (Supplementary Figure S8D). Malignant cells showed higher mean ANGPTL4 expression, with a significant cell-level distributional difference (Supplementary Figure S8E). Among six eligible postoperative patients, malignant-cell ANGPTL4 expression was not significantly correlated with macrophage NLRP3, IL1B, CASP1, or GSDMD (Supplementary Figure S8F).

### 3.4. ANGPTL4 Depletion and Overexpression Alter Proliferative, Migratory, and SA-β-Gal Positivity in LUAD Cells

**Figure 6:**
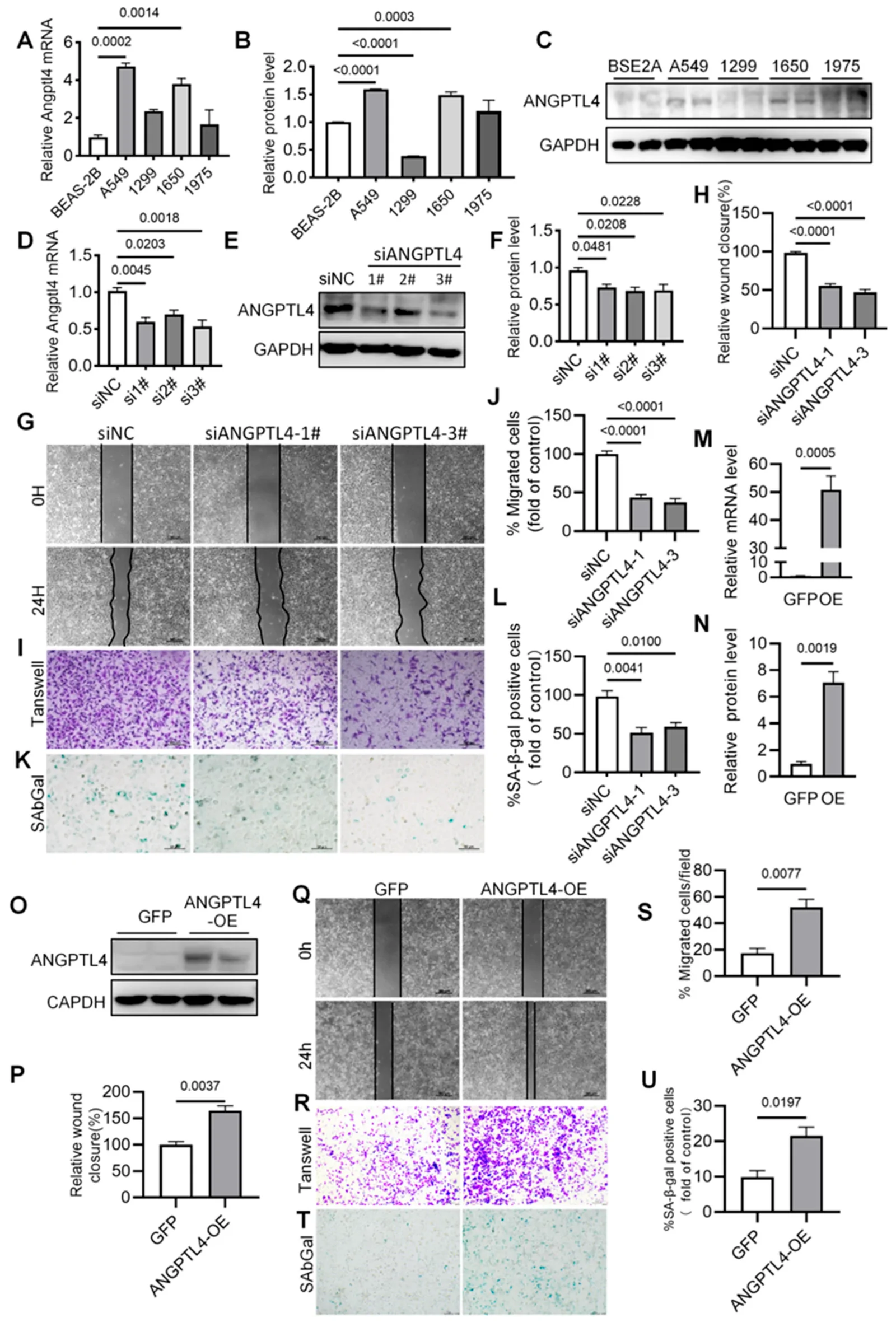
Effects of ANGPTL4 knockdown and overexpression on A549-cell proliferation, migration, and SA-β-gal positivity. (**A**) RT-PCR and (**B**,**C**) Western blot analysis and qualification of ANGPTL4 expression in different LUAD cell lines. (**D**) RT-PCR and (**E**,**F**) Western blot validation of ANGPTL4 knockdown efficiency in A549 cells transfected with si-ANGPTL4, *n* = 3. (**G**,**H**) Wound healing assays showing impaired migratory ability after ANGPTL4 silencing, *n* = 3, scale bar, 50 μm. (**I**,**J**) Transwell migration assays demonstrating reduced migration following ANGPTL4 knockdown, *n* = 4, scale bar, 100 μm. (**K**,**L**) SA-β-gal staining showing a lower proportion of SA-β-gal-positive cells following ANGPTL4 depletion, *n* = 3, scale bar, 200 μm. (**M**) RT-PCR and (**N**,**O**) Western blot validation of stable ANGPTL4 overexpression in A549 cells. (**P**,**Q**) Wound healing (scale bar, 200 μm) and (**R**,**S**) Transwell migration assays (scale bar, 100 μm) demonstrating increased migratory capacity (*n* = 3). (**T**,**U**) SA-β-gal staining showing a higher proportion of SA-β-gal-positive cells following ANGPTL4 overexpression (*n* = 3), scale bar, 100 μm.

We next examined the effects of ANGPTL4 manipulation in LUAD cell models. ANGPTL4 expression was evaluated in several lung cancer cell lines, and relatively higher expression was observed in A549 and H1650 cells (Figure 6A–C). These two cell lines were therefore selected for subsequent experiments. siRNA-mediated depletion reduced ANGPTL4 mRNA and protein expression in A549 cells (Figure 6D–F). ANGPTL4 depletion was accompanied by reduced migratory capacity in both wound-healing and Transwell assays (Figure 6G–J). The proportion of SA-β-gal-positive cells was also lower after ANGPTL4 silencing (Figure 6K–L).

Conversely, stable ANGPTL4-overexpressing A549 cells were generated by lentiviral transduction, and increased ANGPTL4 expression was confirmed by RT-qPCR and Western blotting (Figure 6M–O). ANGPTL4 overexpression was associated with enhanced cell proliferation (Supplementary Figure S9). Increased migration was also observed in wound-healing and Transwell assays (Figure 6P–S). In addition, ANGPTL4-overexpressing cells showed a higher proportion of SA-β-gal-positive cells (Figure 6T,U).

Similar changes in migration, proliferation-associated readouts, and SA-β-gal positivity were observed in H1650 cells after ANGPTL4 depletion or overexpression (Supplementary Figures S10 and S11). Together, these experiments show that ANGPTL4 manipulation alters LUAD-cell migration, proliferation and SA-β-gal positivity. Because senescence was assessed primarily by SA-β-gal staining in these experiments, the results are interpreted as senescence-like or senescence-associated phenotypic changes rather than evidence of a complete canonical senescence program.

### 3.5. ANGPTL4 Overexpression Increases LUAD Xenograft Growth

**Figure 7:**
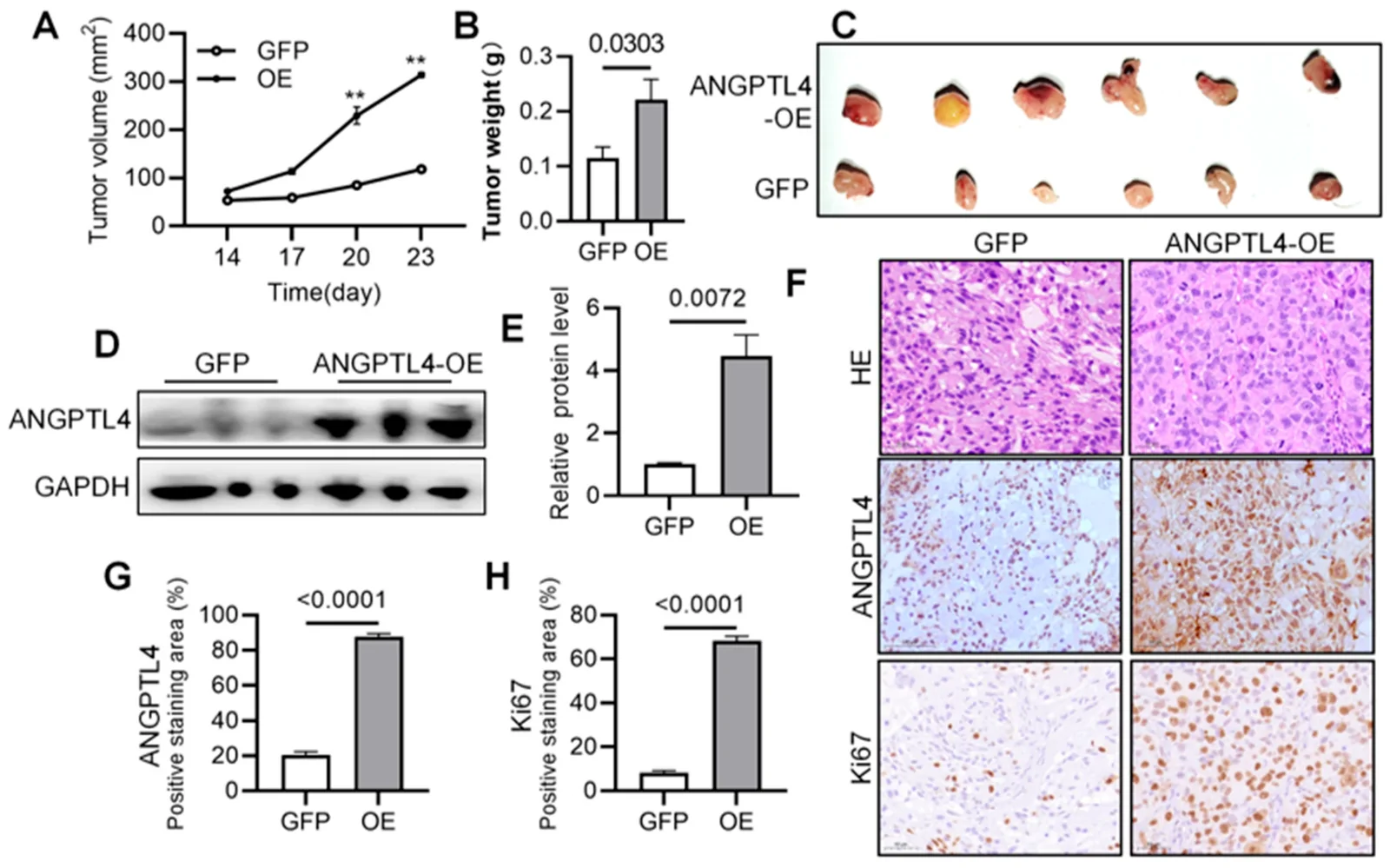
ANGPTL4 overexpression increases A549 xenograft growth. (**A**) Tumor growth curves and (**B**) final tumor weight measured at the endpoint of the experiment (*n* = 6). (**C**) Representative images of subcutaneous tumors excised from nude mice in the control and ANGPTL4-overexpression groups. (**D**,**E**) Western blot and quantification of ANGPTL4 in mice tumors (*n* = 3). (**F**) Representative HE and IHC images showing increased ANGPTL4 and Ki-67 expression in ANGPTL4-overexpressing xenograft tumors, scale bar, 50 μm. (**G**,**H**) Quantification of ANGPTL4 and Ki-67-positive cells in tumor sections (*n* = 3). Data are presented as mean ± SEM. ** *p* < 0.01.

To determine whether the in vitro phenotype was also observed in vivo, A549 cells stably overexpressing ANGPTL4 or corresponding vector-control cells were injected subcutaneously into nude mice. Tumors derived from ANGPTL4-overexpressing cells showed larger tumor volumes and higher final tumor weights than control tumors (Figure 7A–C). Increased ANGPTL4 expression in xenograft tissues was confirmed by Western blotting and immunohistochemistry (Figure 7D–G). Ki-67 staining showed higher proliferative activity in tumors derived from ANGPTL4-overexpressing cells (Figure 7F,H). These findings support an association between elevated ANGPTL4 expression and increased LUAD xenograft growth in this model.

### 3.6. ANGPTL4-Overexpressing LUAD Cells Induce Macrophage Inflammatory Changes Associated with NLRP3 Signaling

**Figure 8:**
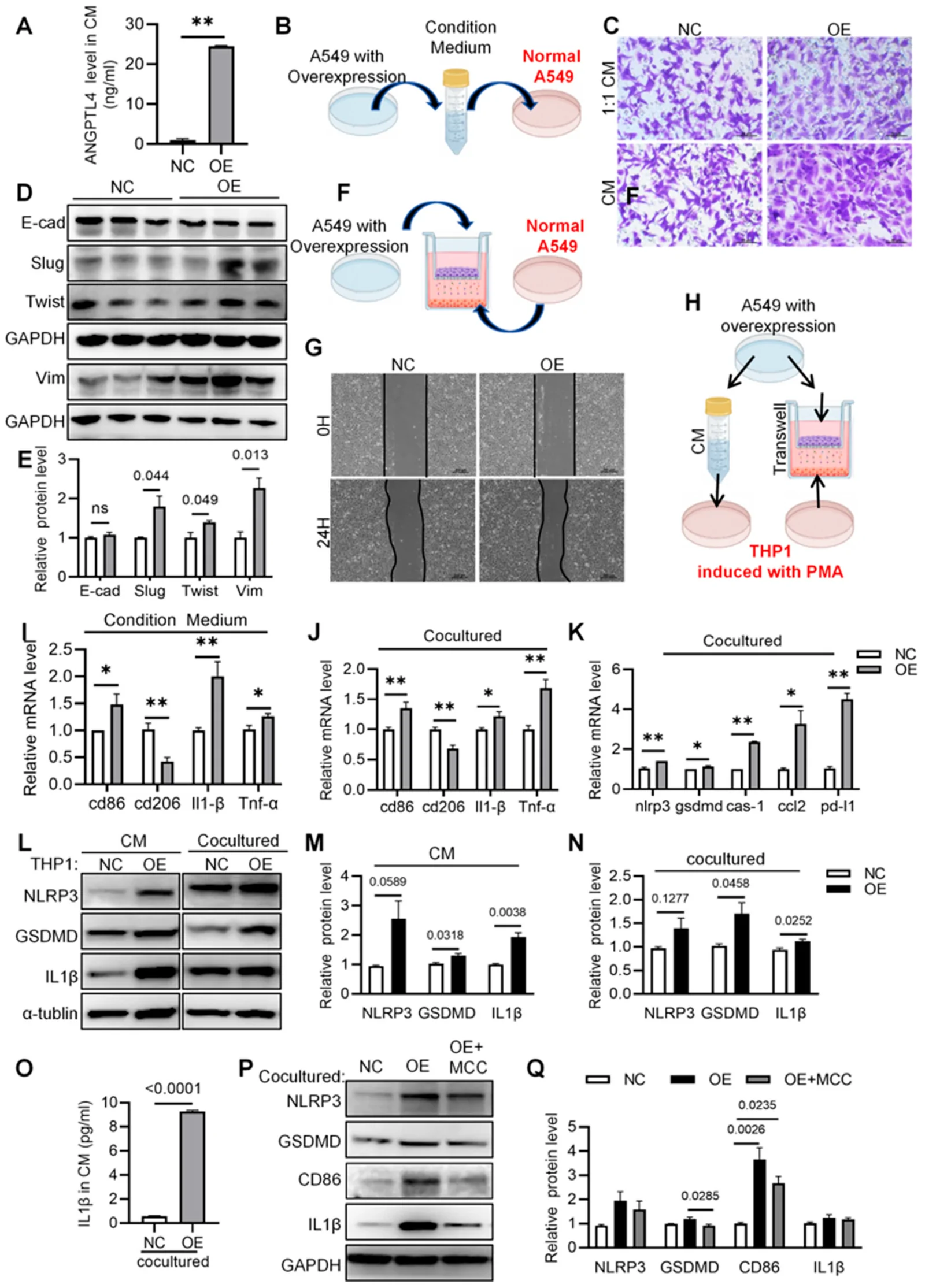
Effects of ANGPTL4-overexpressing A549 cells on recipient tumor-cell migration and macrophage inflammatory responses. (**A**) ELISA analysis of ANGPTL4 secretion levels in CM collected from control and ANGPTL4-overexpressing A549 cells (*n* = 3). (**B**) Schematic illustration of the CM treatment assay, in which native A549 cells were treated with CM derived from control or ANGPTL4-overexpressing A549 cells. (**C**) Transwell migration assays treated with undiluted and 1:1 diluted CM, scale bar, 50 μm. (**D**,**E**) Western blot analysis and qualification of EMT-related markers in A549 cells treated with CM (*n* = 3). (**F**) Schematic illustration of the indirect co-culture system between native A549 cells and ANGPTL4-overexpressing A549 cells. (**G**) Wound healing assays, scale bar, 200 μm. (**H**) Schematic illustration of conditioned-medium treatment and indirect co-culture systems used to assess THP-1-derived macrophage responses to ANGPTL4-overexpressing LUAD cells and their secretome. (**I**,**J**) Quantitative analysis showing changes in macrophage polarization markers in CM-treated or co-cultured macrophages (*n* = 3). (**K**) qRT-PCR analysis of the expression of NLRP3 inflammasome-related genes (*nlrp3, gsdmd, caspase-1*) and pro-tumor factors (*ccl2, pd-l1*) in macrophages *(n* = 3). (**L**–**N**) Western blot analysis and qualification of NLRP3, GSDMD, and IL-1β in macrophages exposed to ANGPTL4-overexpressing cells or their CM (*n* = 3). (**O**) ELISA analysis IL-1β in co-culture CM (*n* = 4). (**P**,**Q**) Western blot analysis and qualification of NLRP3, GSDMD, IL-1β, and CD86 in THP-1-derived macrophages exposed to conditioned medium from ANGPTL4-overexpressing LUAD cells, with or without MCC950 treatment (*n* = 3). Data are presented as mean ± SEM. * *p* < 0.05, ** *p* < 0.01.

To explore the effects of the secretome of ANGPTL4-overexpressing LUAD cells, we examined whether conditioned medium from ANGPTL4-overexpressing LUAD cells affected neighboring tumor cells and macrophages. ELISA confirmed increased ANGPTL4 concentrations in conditioned medium collected from ANGPTL4-overexpressing A549 cells compared with control cells (Figure 8A). Conditioned medium from ANGPTL4-overexpressing cells increased the migration of untransfected A549 cells in Transwell assays, both when used undiluted and after 1:1 dilution (Figure 8B,C). This treatment was accompanied by reduced E-cadherin and increased Vimentin, Slug, and Twist expression, consistent with an EMT-associated expression pattern (Figure 8D,E). An indirect co-culture system containing naïve A549 cells and ANGPTL4-overexpressing A549 cells produced a similar increase in migratory capacity (Figure 8F,G).

We then examined the effects of the tumor-cell secretome on THP-1-derived macrophages. Macrophages exposed to conditioned medium from ANGPTL4-overexpressing cells or indirectly co-cultured with these cells showed reduced expression of the M2-associated marker CD206 and increased the mRNA levels of inflammatory-associated markers, including CD86, IL-1β, and TNF-α (Figure 8H–J). The expression of NLRP3-associated genes, including NLRP3, GSDMD, and Caspase-1, as well as CCL2 and PD-L1, was also increased in macrophages exposed to the ANGPTL4-overexpressing tumor-cell secretome (Figure 8K). Western blotting confirmed higher NLRP3, GSDMD, and IL-1β protein levels in macrophages exposed to conditioned medium from ANGPTL4-overexpressing cells (Figure 8L–N). Consistently, ELISA demonstrated increased IL-1β secretion in conditioned medium collected from the co-culture system (Figure 8O).

To assess whether NLRP3-associated signaling contributed to these macrophage responses, macrophages were pretreated with the NLRP3 inhibitor MCC950. MCC950 partly reduced the increases in NLRP3, GSDMD, IL-1β, and CD86 associated with exposure to the ANGPTL4-overexpressing-cell conditioned medium (Figure 8P,Q). Together, these data associate the secretome of ANGPTL4-overexpressing LUAD cells with macrophage inflammatory remodeling, while the specific contribution of ANGPTL4 remains unresolved.

## 4. Discussion

The present study prioritized ANGPTL4 through senescence-associated transcriptomic analyses and examined its relationship with LUAD-cell phenotypes and macrophage responses to the tumor-cell secretome. By integrating bulk transcriptomic analysis, single-cell RNA sequencing, clinical sample analysis, and functional experiments, we found that senescence-associated transcriptional alterations in LUAD were linked to inflammatory and immune-related features. Single-cell analysis further localized higher senescence-associated gene-set scores to macrophage- and monocyte-enriched compartments and revealed increased intercellular communication in the higher-score group. In addition, ANGPTL4 was upregulated in LUAD tissues and promoted tumor-cell proliferation, migration, changes in SA-β-gal positivity, and xenograft growth. Conditioned medium from ANGPTL4-overexpressing cells also induced macrophage inflammatory changes that were attenuated by MCC950.

The single-cell findings require cautious interpretation. SenMayo contains inflammatory and secretory genes that can be expressed constitutively by myeloid cells; high scores may therefore reflect cell identity or activation rather than senescence. The dataset contains both LUAD and squamous carcinomas and includes treatment-related variation. The epithelial comparison does not establish malignant cells as the dominant ANGPTL4 source across all cell types, nor does it establish an association between ANGPTL4 and high versus low senescence scores within malignant cells. Furthermore, the six-patient macrophage correlation analysis was small and yielded no significant associations. These observations limit the single-cell evidence to descriptive context and do not directly validate ANGPTL4-mediated macrophage signaling.

Cellular senescence is traditionally viewed as a tumor-suppressive response because stable cell-cycle arrest limits the expansion of damaged or oncogene-stressed cells. However, senescence is heterogeneous, and its effects depend on cellular context, duration, and secretory composition [10,11,12,13]. Senescent cells can influence neighboring epithelial, stromal, and immune cells by releasing cytokines, chemokines, growth factors, and matrix-remodeling molecules [9,12]. Therapy-induced senescence may also promote cancer stemness and tumor relapse [25], although these processes were not examined here. In our study, ANGPTL4 manipulation changed SA-β-gal positivity while also affecting proliferation and migration. These findings should therefore be interpreted as evidence of senescence-associated or senescence-like features rather than a complete canonical senescence program. This interpretation is consistent with the absence of a single definitive senescence marker and the known heterogeneity of tumor-associated senescence states. Because these endpoints were assessed in separate assays, the proliferative status of individual SA-β-gal-positive cells remains unknown. Increased lysosomal activity, differences in cell density, or heterogeneous cell states could contribute to the staining results, but these possibilities were not directly tested. Without evidence of stable growth arrest and additional independent markers, the findings do not establish a defined senescent cell population.

A recent study showed that senescent synovial fibroblasts promoted inflammatory macrophage responses through paracrine ANGPTL4 signaling [26], supporting a possible role for ANGPTL4 in senescence-associated intercellular communication. Consistent with this possibility, conditioned medium from ANGPTL4-overexpressing LUAD cells increased the migration of neighboring tumor cells and was accompanied by an EMT-associated expression pattern. Chronic inflammatory signaling can reinforce SASP-related remodeling and create a tumor-permissive microenvironment [27]. Thus, conditioned medium from ANGPTL4-overexpressing cells affected both recipient tumor-cell migration and macrophage inflammatory markers.

NLRP3 is an innate immune sensor that regulates caspase-1 activation and the maturation of inflammatory cytokines, including IL-1β and IL-18 [28]. Dysregulated NLRP3 signaling has been implicated in chronic inflammation and cancer progression [29], and inflammasome-related pathways have also been associated with cellular stress and senescence-related responses [30]. In our experiments, the secretome of ANGPTL4-overexpressing LUAD cells increased inflammatory markers and NLRP3-associated proteins in THP-1-derived macrophages. These changes were attenuated by MCC950, supporting a contribution of NLRP3 signaling to the macrophage response. However, our findings do not demonstrate that ANGPTL4 directly activates NLRP3 and the precise molecular components responsible for this response therefore remain to be defined.

## 5. Conclusions

In conclusion, ANGPTL4 overexpression increased LUAD-cell proliferation, migration, and xenograft growth and altered SA-β-gal positivity. Conditioned medium from ANGPTL4-overexpressing cells induced macrophage inflammatory changes, some of which were attenuated by MCC950. However, several limitations constrain these findings. Senescence-associated gene-set scores and SA-β-gal staining did not establish stable growth arrest or a defined senescent state. Validation in additional LUAD models with low endogenous ANGPTL4 expression is also needed. The conditioned-medium experiments did not isolate the contribution of extracellular ANGPTL4 from that of other secreted factors. Macrophage responses were assessed only in THP-1-derived cells, without validation in primary peripheral blood monocytes or monocyte-derived macrophages from independent donors. The reciprocal effects of immune-cell-conditioned medium on LUAD cells were also not examined. Finally, the xenograft experiments did not determine whether macrophages or NLRP3 signaling mediated the observed tumor growth.

## Data Availability

The datasets supporting the conclusions of this article are available from the corresponding author upon reasonable request.

## Supplementary Materials

The following supporting information can be downloaded at: https://www.mdpi.com/article/10.3390/cancers18213571/s1, Figure S1: Survival stratification and time-dependent ROC validation across cohorts; Figure S2: GSVA and GSEA enrichment analyses for high- and low-risk groups; Figure S3: Overview of immune modulators, drug sensitivity, and immunotherapy-related indicators; Figure S4: Immune infiltration profiles and ESTIMATE score evaluation; Figure S5: Univariate and multivariable Cox analyses of overall survival in TCGA-LUAD. Figure S6: Quality control and batch-effect correction of single-cell data; Figure S7: Cell-cell communication analysis between high- and low-senescence groups; Figure S8: Inferred epithelial malignancy and exploratory ANGPTL4 analyses. Figure S9: ANGPTL4 knockdown inhibits LUAD cell proliferation, migration, and SA-β-gal positivity in H1650 cells. Figure S10: ANGPTL4 knockdown inhibits LUAD Cell Proliferation, Migration, and SA-β-gal positivity In Vitro; Figure S11: ANGPTL4 overexpression promotes LUAD cell proliferation, migration, and SA-β-gal positivity in H1650 cells; File S1: the original western blots.

## References

[1] Herbst R.S., Morgensztern D., Boshoff C. (2018). The biology and management of non-small cell lung cancer. Nature.

[2] Sung H., Ferlay J., Siegel R.L., Laversanne M., Soerjomataram I., Jemal A., Bray F. (2021). Global Cancer Statistics 2020: GLOBOCAN Estimates of Incidence and Mortality Worldwide for 36 Cancers in 185 Countries. CA Cancer J. Clin..

[3] Hanahan D. (2026). Hallmarks of cancer-Then and now, and beyond. Cell.

[4] Lambrechts D., Wauters E., Boeckx B., Aibar S., Nittner D., Burton O., Bassez A., Decaluwé H., Pircher A., Van den Eynde K. (2018). Phenotype molding of stromal cells in the lung tumor microenvironment. Nat. Med..

[5] Cassetta L., Fragkogianni S., Sims A.H. (2019). Human Tumor-Associated Macrophage and Monocyte Transcriptional Landscapes Reveal Cancer-Specific Reprogramming, Biomarkers, and Therapeutic Targets. Cancer Cell.

[6] Sun X., Park M.D., Merad M., Brown B.D. (2026). Macrophages: Targets for next-generation cancer immunotherapy. Cancer Cell.

[7] Cassetta L., Pollard J.W. (2018). Targeting macrophages: Therapeutic approaches in cancer. Nat. Rev. Drug Discov..

[8] Gorgoulis V., Adams P.D., Alimonti A., Bennett D.C., Bischof O., Bishop C., Campisi J., Collado M., Evangelou K., Ferbeyre G. (2019). Cellular Senescence: Defining a Path Forward. Cell.

[9] Faget D.V., Ren Q., Stewart S.A. (2019). Unmasking senescence: Context-dependent effects of SASP in cancer. Nat. Rev. Cancer.

[10] Basisty N., Kale A., Jeon O.H., Kuehnemann C., Payne T., Rao C., Holtz A., Shah S., Sharma V., Ferrucci L. (2020). A proteomic atlas of senescence-associated secretomes for aging biomarker development. PLoS Biol..

[11] Colucci M., Sarill M., Maddalena M., Valdata A., Troiani M., Massarotti M., Bolis M., Bressan S., Kohl A., Robesti D. (2025). Senescence in cancer. Cancer Cell.

[12] Wang B., Han J., Elisseeff J.H., Demaria M. (2024). The senescence-associated secretory phenotype and its physiological and pathological implications. Nat. Rev. Mol. Cell Biol..

[13] Coppé J.P., Desprez P.Y., Krtolica A., Campisi J. (2010). The senescence-associated secretory phenotype: The dark side of tumor suppression. Annu. Rev. Pathol..

[14] Zilionis R., Engblom C., Pfirschke C., Savova V., Zemmour D., Saatcioglu H.D., Krishnan I., Maroni G., Meyerovitz C.V., Kerwin C.M. (2019). Single-Cell Transcriptomics of Human and Mouse Lung Cancers Reveals Conserved Myeloid Populations across Individuals and Species. Immunity.

[15] Aryal B., Price N.L., Suarez Y., Fernández-Hernando C. (2019). ANGPTL4 in Metabolic and Cardiovascular Disease. Trends Mol. Med..

[16] Ramos P., Shi Q., Kleberg J., Maharjan C.K., Zhang W., Kolb R. (2025). ANGPTL4: A Comprehensive Review of 25 Years of Research. Cancers.

[17] Xu J., Wu F., Zhu Y., Wu T., Cao T., Gao W., Liu M., Qian W., Feng G., Xi X. (2024). ANGPTL4 regulates ovarian cancer progression by activating the ERK1/2 pathway. Cancer Cell Int..

[18] Liu R., Fu M., Chen P., Liu Y., Huang W., Sun X., Zhu P., Wen Z., Cheng Y. (2024). Emerging roles of angiopoietin-like 4 in human tumors (Review). Int. J. Oncol..

[19] Xiao S., Nai-Dong W., Jin-Xiang Y., Long T., Xiu-Rong L., Hong G., Jie-Cheng Y., Fei Z. (2022). ANGPTL4 regulate glutamine metabolism and fatty acid oxidation in nonsmall cell lung cancer cells. J. Cell. Mol. Med..

[20] Zhang Y., Liu X., Zeng L., Zhao X., Chen Q., Pan Y., Bai Y., Shao C., Zhang J. (2022). Exosomal protein angiopoietin-like 4 mediated radioresistance of lung cancer by inhibiting ferroptosis under hypoxic microenvironment. Br. J. Cancer.

[21] Zhu X., Guo X., Wu S., Wei L. (2016). ANGPTL4 Correlates with NSCLC Progression and Regulates Epithelial-Mesenchymal Transition via ERK Pathway. Lung.

[22] Wang X., Chang H.C., Gu X., Zhang Y., Bao Z. (2025). Adipose Factor ANGPTL4: Its Role in Aging Mechanisms and Associated Diseases. Clin. Interv. Aging.

[23] Saul D., Kosinsky R.L., Atkinson E.J., Doolittle M.L., Zhang X., LeBrasseur N.K., Pignolo R.J., Robbins P.D., Niedernhofer L.J., Ikeno Y. (2022). A new gene set identifies senescent cells and predicts senescence-associated pathways across tissues. Nat. Commun..

[24] Yan Y., Sun D., Hu J., Chen Y., Sun L., Yu H., Xiong Y., Huang Z., Xia H., Zhu X. (2025). Multi-omic profiling highlights factors associated with resistance to immuno-chemotherapy in non-small-cell lung cancer. Nat. Genet..

[25] Milanovic M., Fan D.N.Y., Belenki D., Däbritz J.H.M., Zhao Z., Yu Y., Dörr J.R., Dimitrova L., Lenze D., Monteiro Barbosa I.A. (2018). Senescence-associated reprogramming promotes cancer stemness. Nature.

[26] Deng M., Jiang Y., Chen Z., Chen K., Cao N., Huang Y., Quan Z., Chen C. (2026). Senescent Synovial Intimal Fibroblasts Aggravate Osteoarthritis by Regulating Macrophage Polarization and Chondrocyte Phenotype Through ANGPTL4-α5β1 Axis. Adv. Sci..

[27] Greten F.R., Grivennikov S.I. (2019). Inflammation and Cancer: Triggers, Mechanisms, and Consequences. Immunity.

[28] Cabral J.E., Wu A., Zhou H., Pham M.A., Lin S., McNulty R. (2025). Targeting the NLRP3 inflammasome for inflammatory disease therapy. Trends Pharmacol. Sci..

[29] Sharma B.R., Kanneganti T.D. (2021). NLRP3 inflammasome in cancer and metabolic diseases. Nat. Immunol..

[30] Shi Y., Zhao L., Wang J., Liu S., Zhang Y., Qin Q. (2022). The selective NLRP3 inflammasome inhibitor MCC950 improves isoproterenol-induced cardiac dysfunction by inhibiting cardiomyocyte senescence. Eur. J. Pharmacol..

