# Supplementary material for: Senescence-Related Transcriptomic Analysis and Functional Characterization of ANGPTL4 in Lung Adenocarcinoma
Source: Cancers (Basel). 2026 Nov 6;18(21):3571. doi: 10.3390/cancers18213571 (PMC13762416; doi:10.3390/cancers18213571)

Figure 3

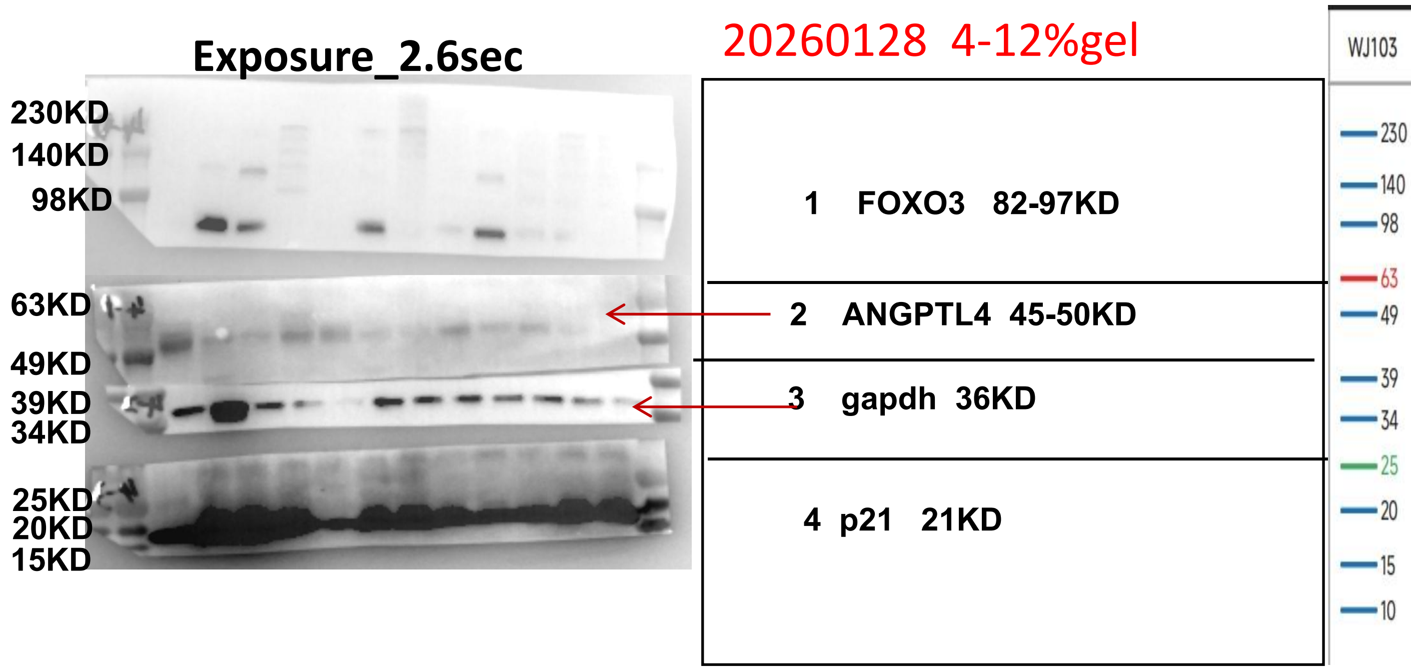

The same band at different exposure time

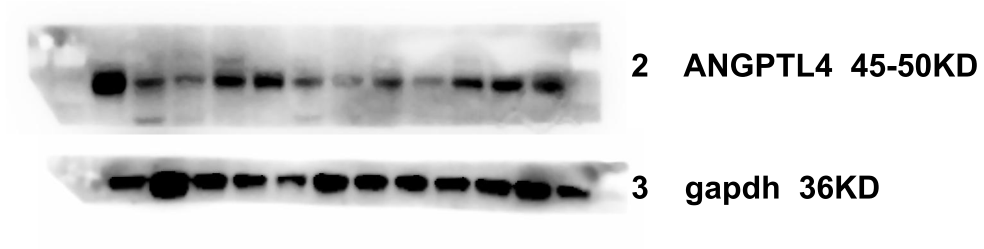

Figure 6C

2025-08-20 _Exposure_1.0sec 7# 20250820 4-12%gel

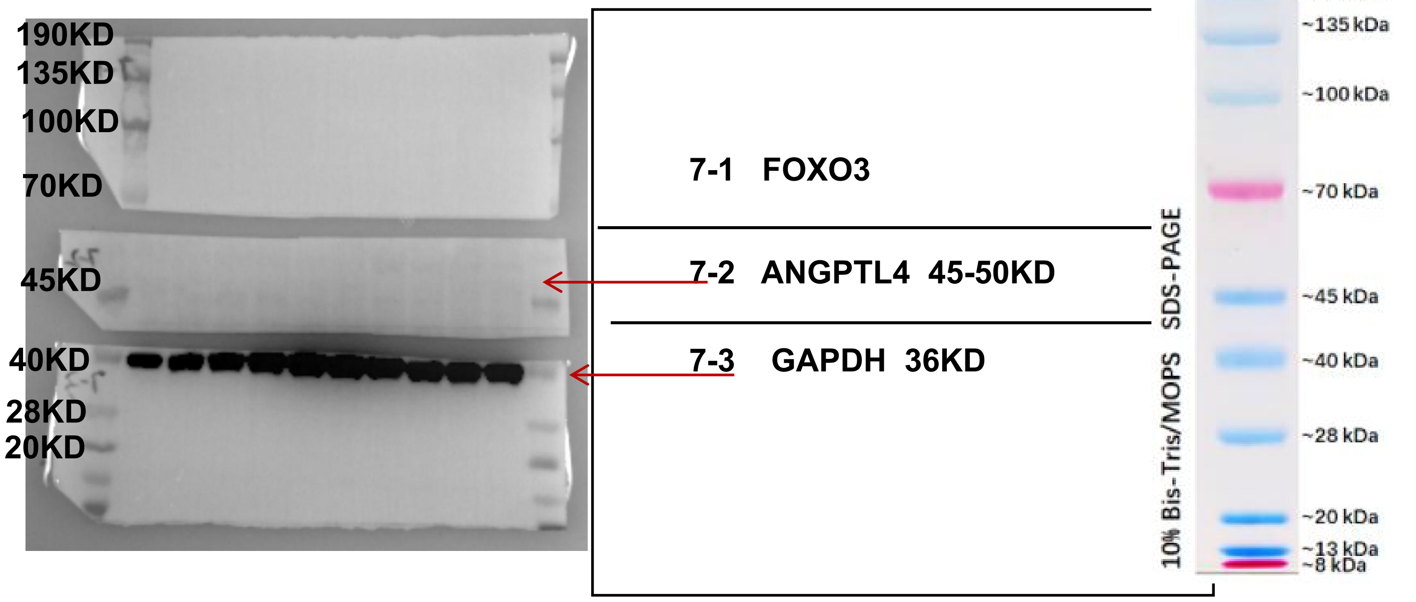

2025-08-20 _Exposure_4.0sec

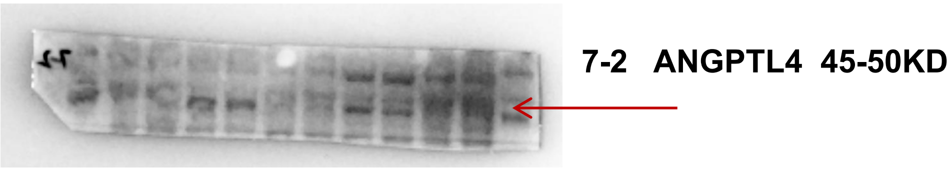

Figure 6E

2025-10-17 _Exposure_3.1sec

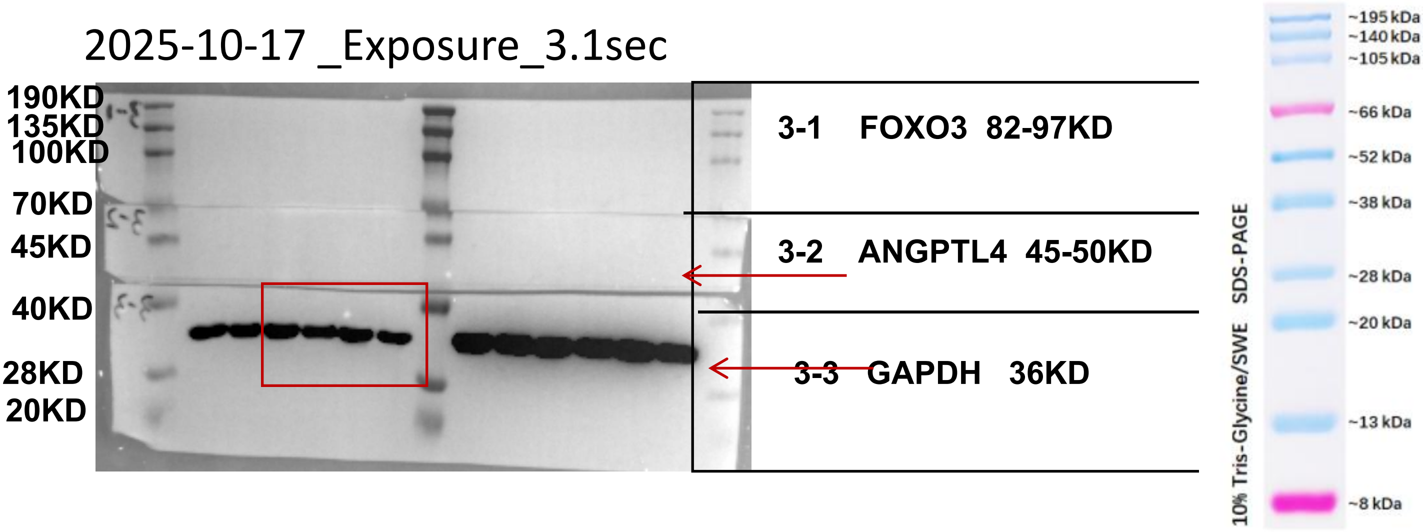

dell 2025-10-17_Exposure_67.4sec

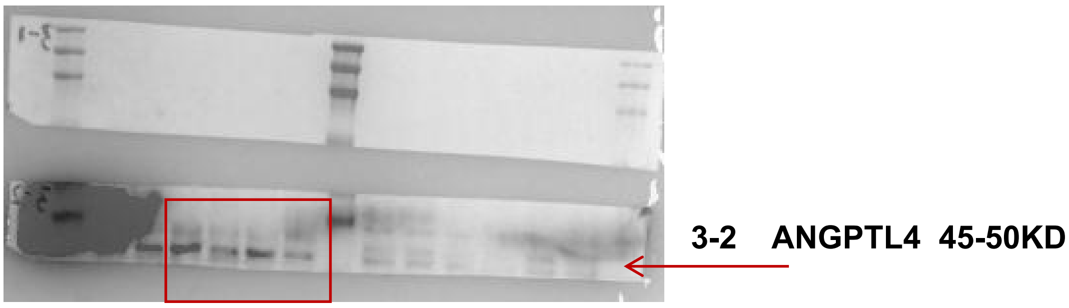

Figure 6O

20260203 4-12%gel

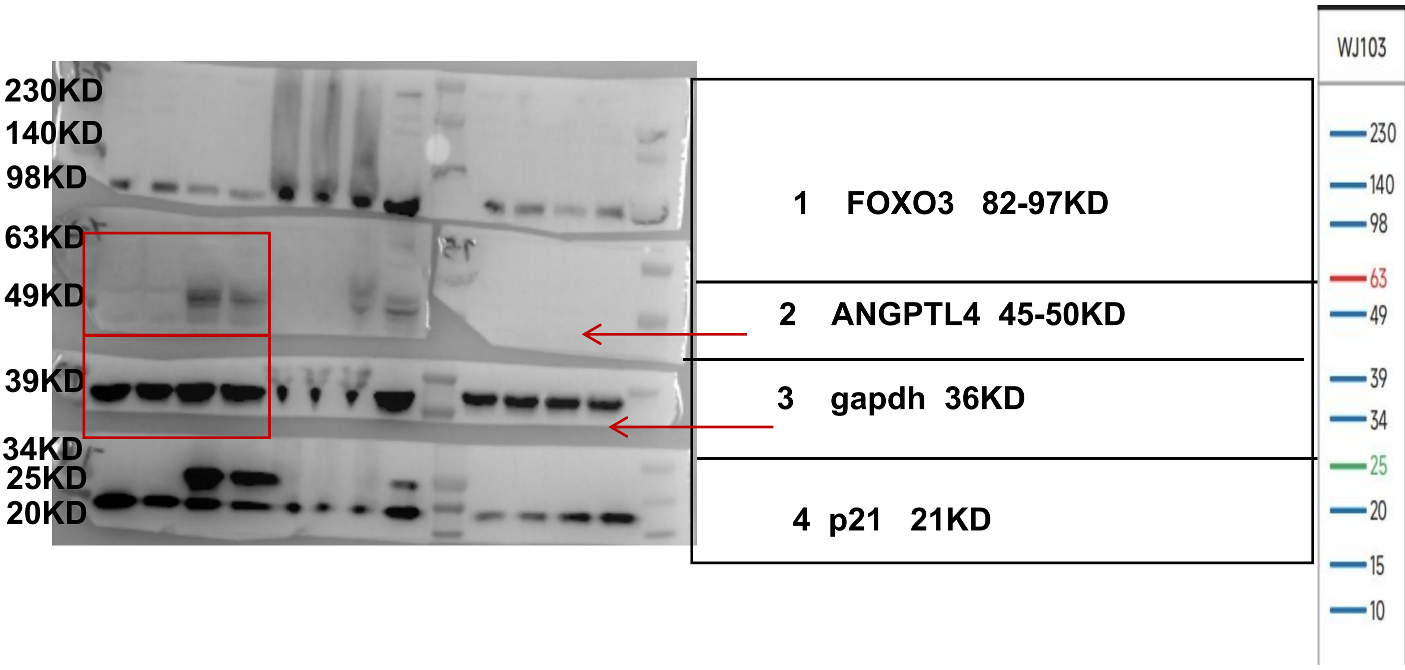

Figure 7D

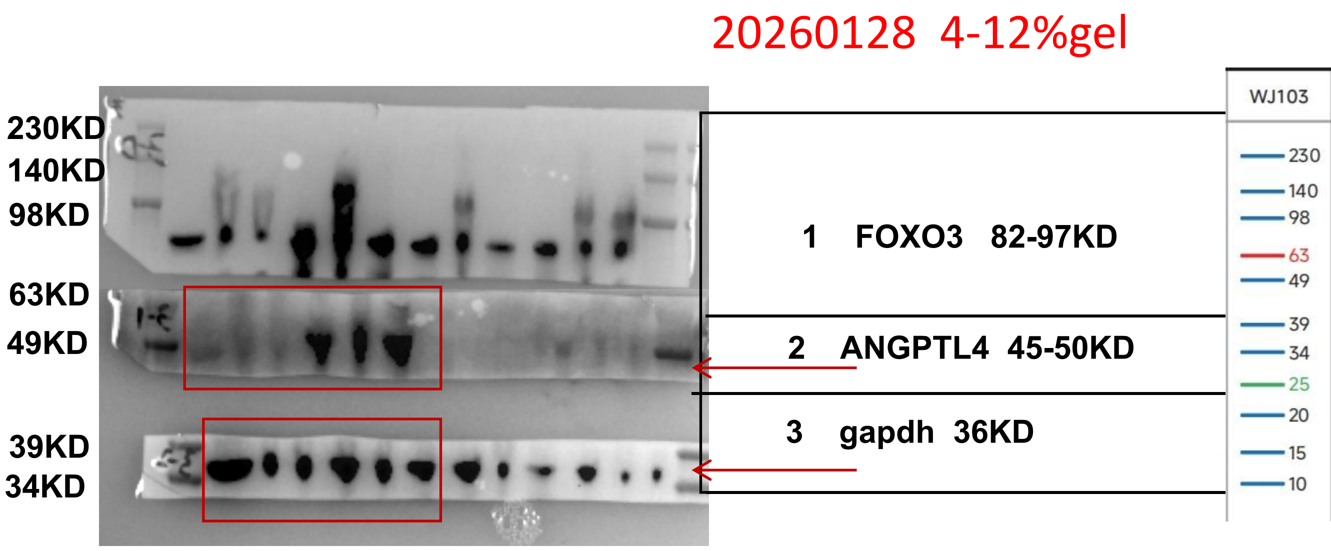
20260128 4-12%gel
230KD
140KD
98KD
63KD
49KD
39KD
34KD
1 FOXO3 82-97KD
2 ANGPTL4 45-50KD
3 gapdh 36KD
WJ103
230
140
98
63
49
39
34
25
20
15
10

Figure 8D

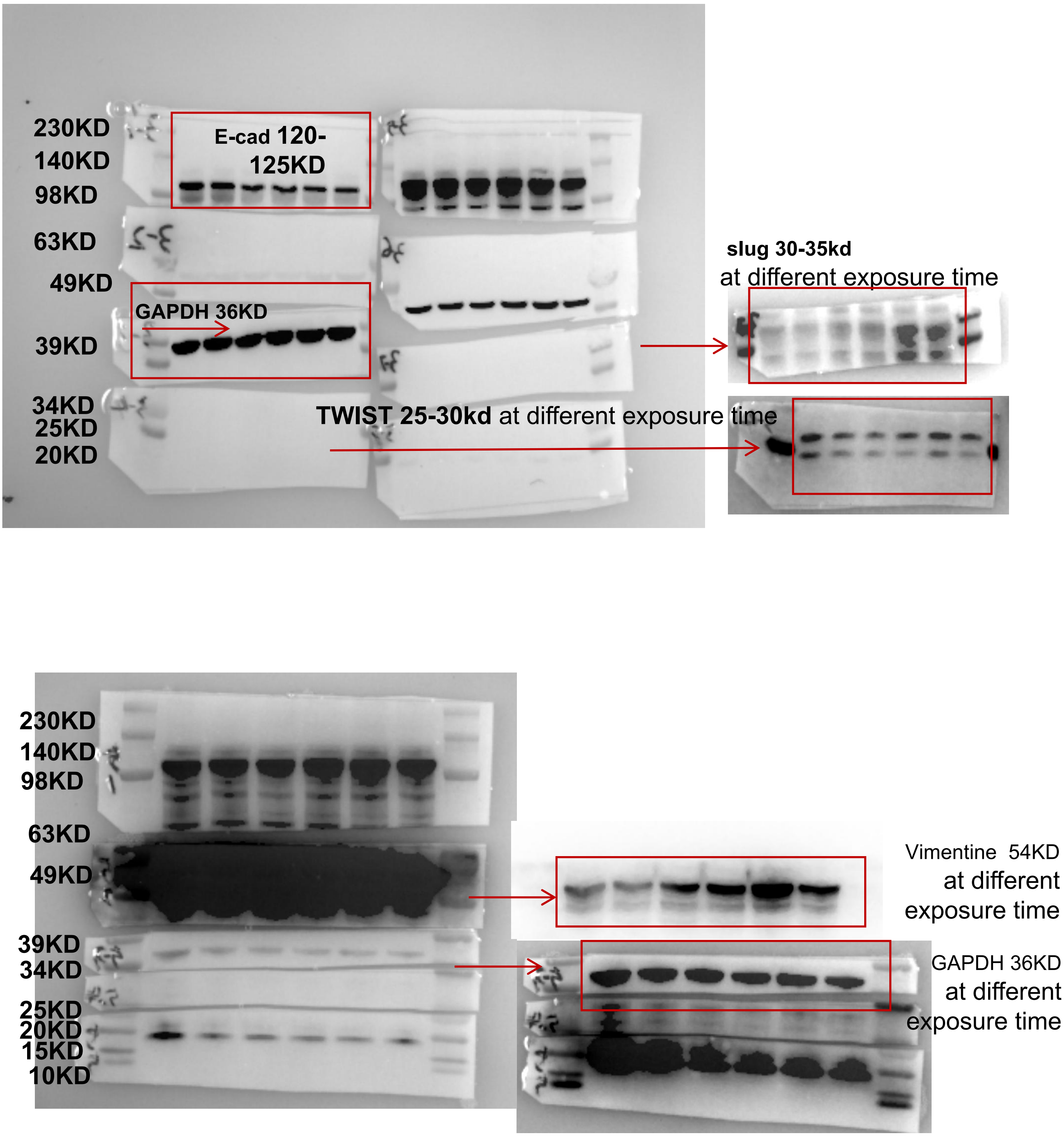
230KD
140KD
98KD
63KD
49KD
39KD
34KD
25KD
20KD
E-cad 120-125KD
GAPDH 36KD
slug 30-35kd
at different exposure time
TWIST 25-30kd at different exposure time
230KD
140KD
98KD
63KD
49KD
39KD
34KD
25KD
20KD
15KD
10KD
Vimentine 54KD
at different exposure time
GAPDH 36KD
at different exposure time

Figure 8L

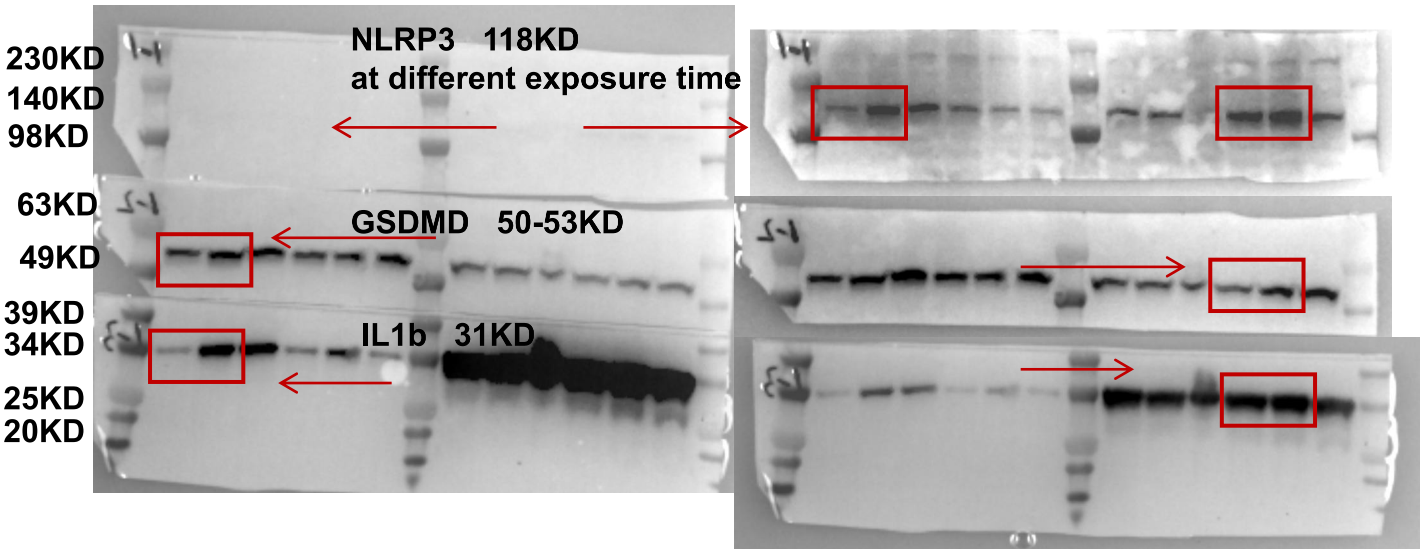
230KD
140KD
98KD
63KD
49KD
39KD
34KD
25KD
20KD
NLRP3 118KD
at different exposure time
GSDMD 50-53KD
IL1b 31KD

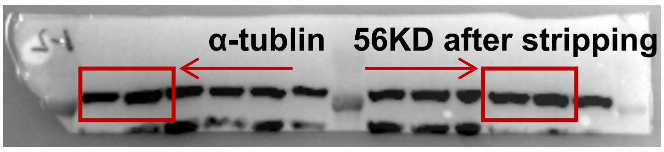
α-tublin 56KD after stripping

Figure 8P

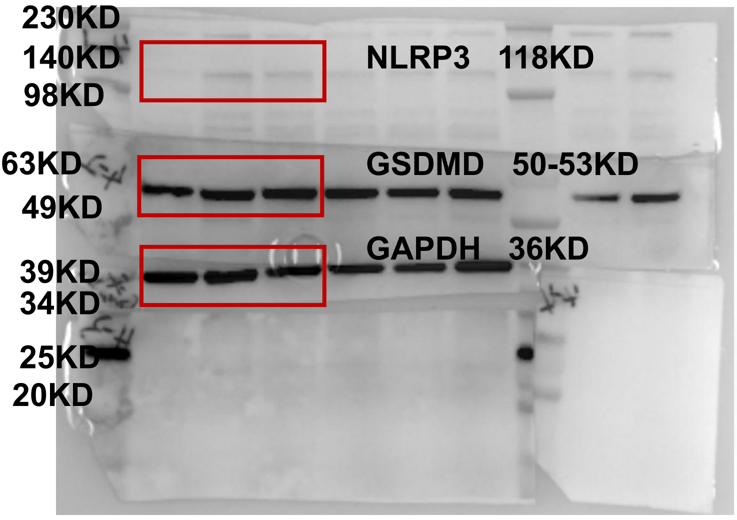
230KD
140KD
98KD
NLRP3 118KD
63KD
49KD
GSDMD 50-53KD
39KD
34KD
GAPDH 36KD
25KD
20KD

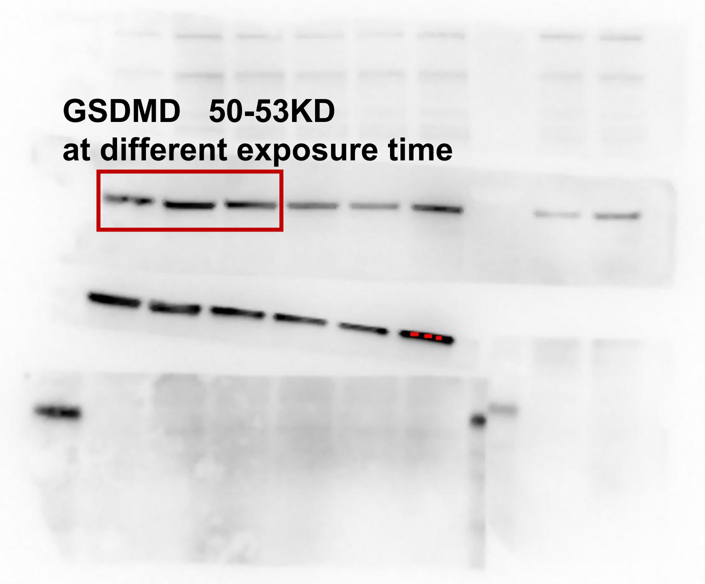
GSDMD 50-53KD
at different exposure time

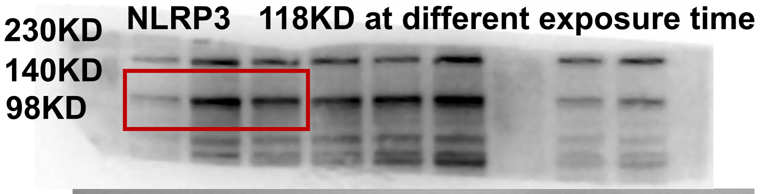
230KD
140KD
98KD
NLRP3 118KD at different exposure time

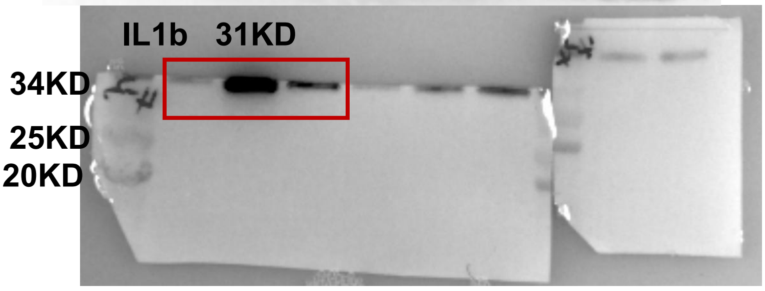
IL1b 31KD
34KD
25KD
20KD

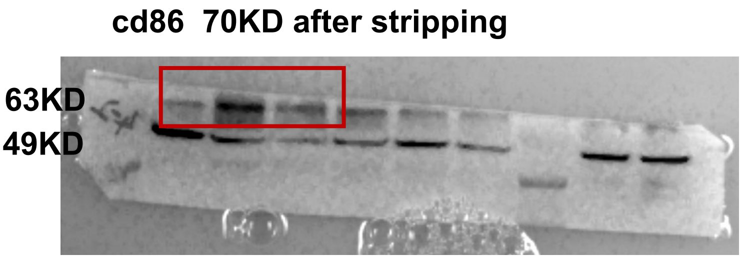
cd86 70KD after stripping
63KD
49KD

Figure S10 B.

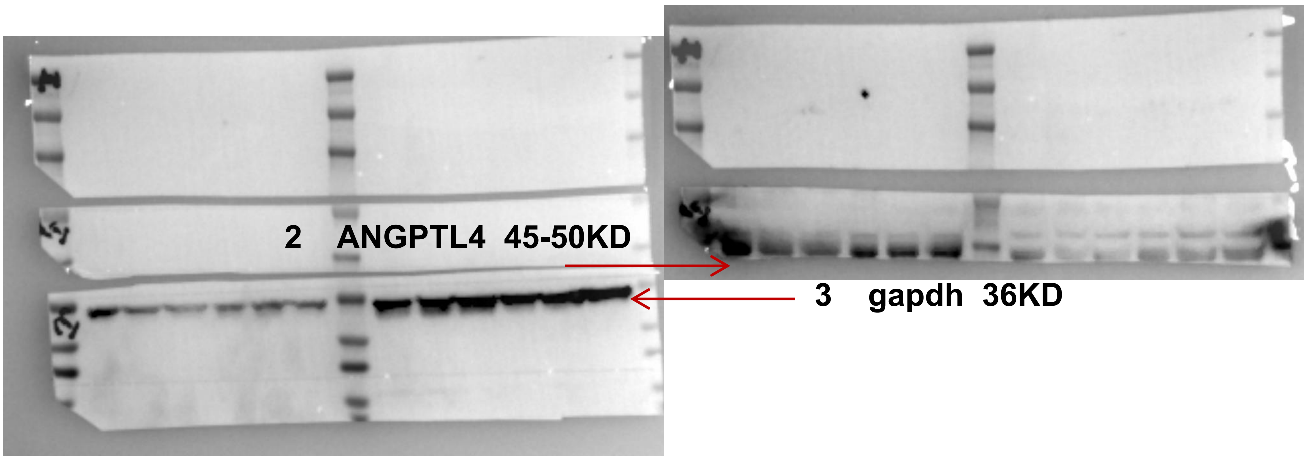

Figure S11 B.

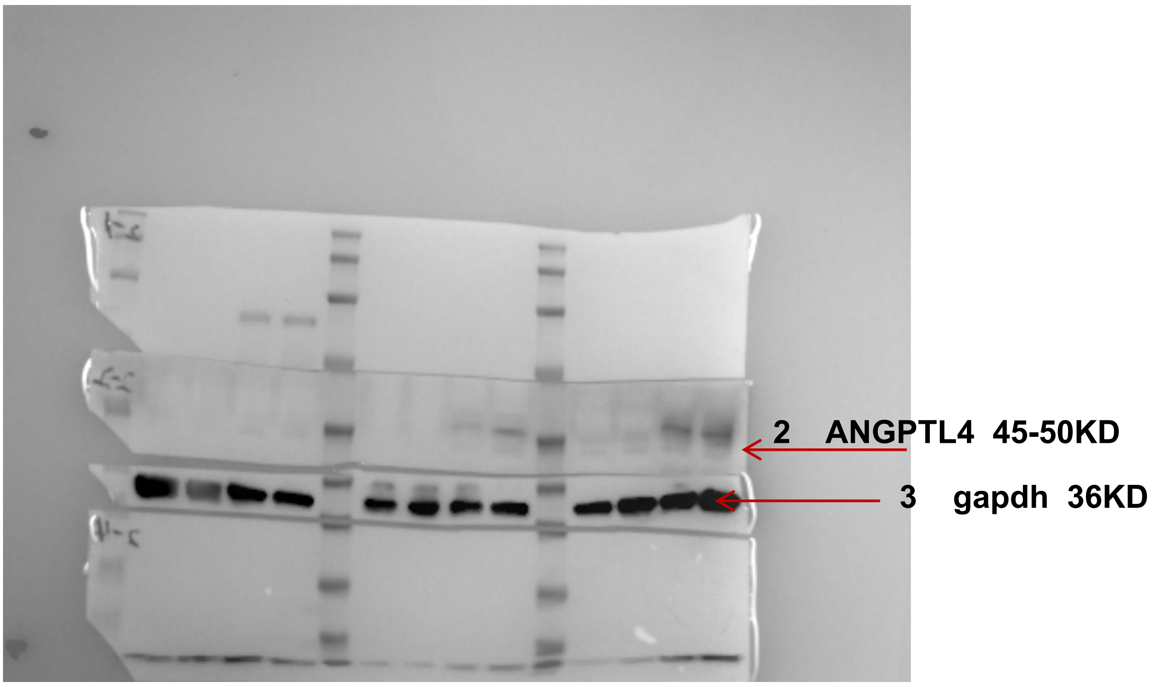

Supplement: Supplementary file 1 [file cancers-18-03571-s001.zip › cancers-4619880-supplementary/cancers-4619880- File S1-original WB.pdf]
