# Supplementary material for: Senescence-Related Transcriptomic Analysis and Functional Characterization of ANGPTL4 in Lung Adenocarcinoma
Source: Cancers (Basel). 2026 Nov 6;18(21):3571. doi: 10.3390/cancers18213571 (PMC13762416; doi:10.3390/cancers18213571)
Supplement: Supplementary file 1 [file cancers-18-03571-s001.zip › cancers-4619880-supplementary/cancers-4619880-supplementary figures.pdf]

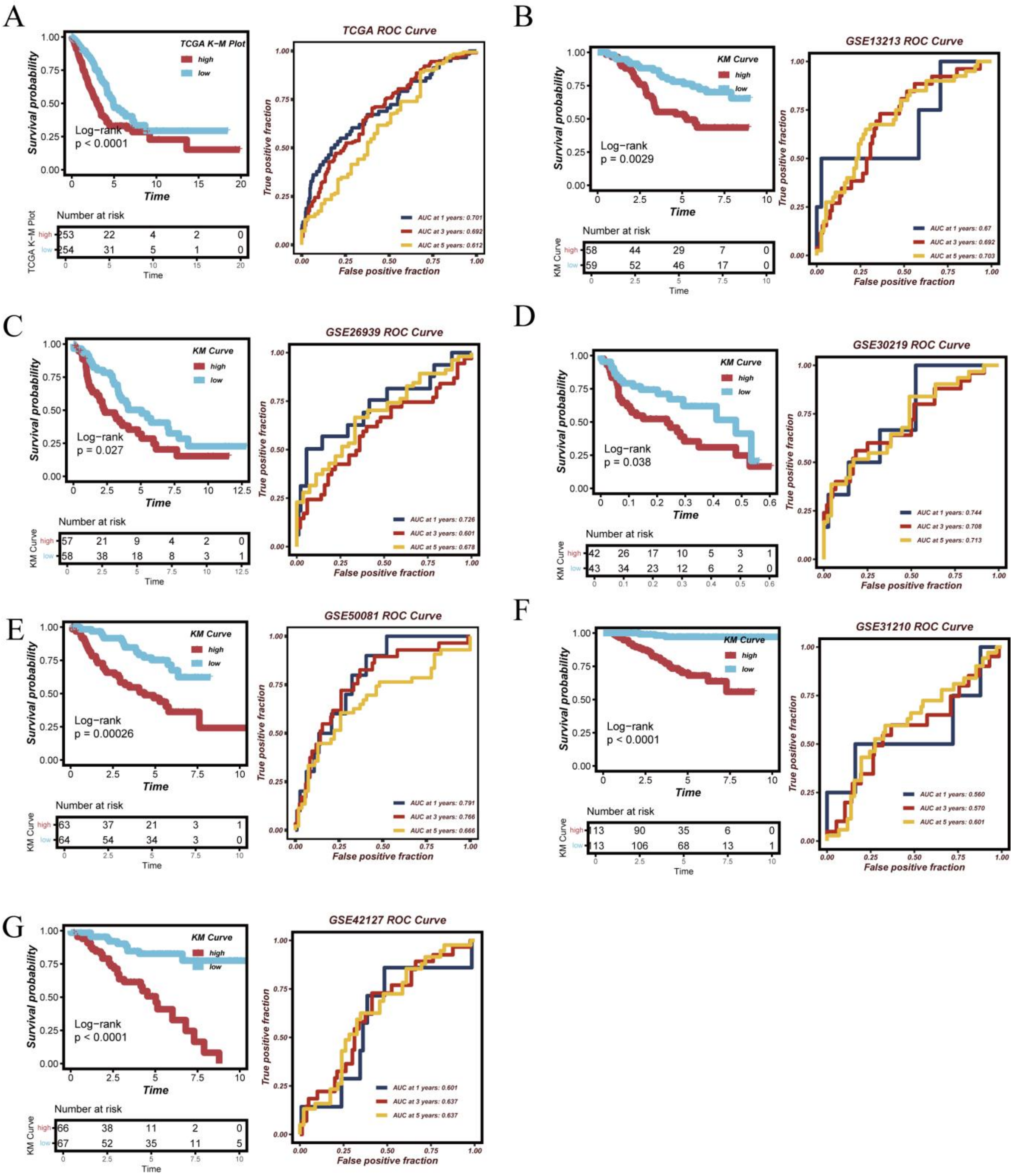

Supplementary Figure S1. Survival stratification and time-dependent ROC analysis. Panels A–G show Kaplan–Meier curves and 1-, 3-, and 5-year ROC curves for TCGA-LUAD (A), GSE13213 (B), GSE26939 (C), GSE30219 (D), GSE50081 (E), GSE31210 (F), and GSE42127 (G). The corresponding 1-/3-/5-year AUCs are 0.701/0.692/0.612, 0.670/0.692/0.703, 0.726/0.601/0.678, 0.744/0.708/0.713, 0.791/0.766/0.666, 0.560/0.570/0.601, and 0.601/0.637/0.637, respectively. Kaplan–Meier p-values are from log-rank tests; numbers at risk are displayed below the survival curves.

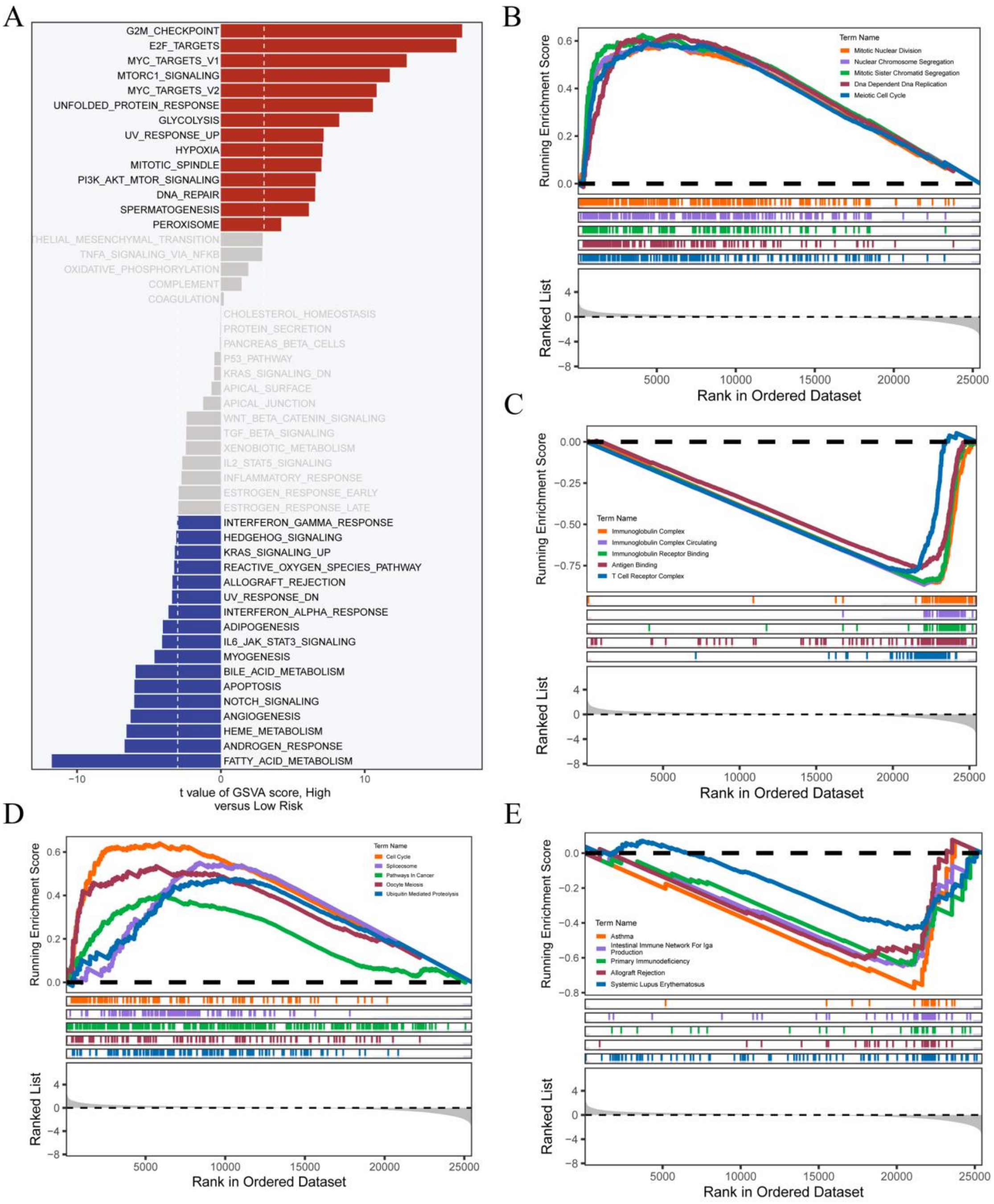

Figure S2. GSVA and GSEA enrichment analyses for high- and low-risk groups. (A) GSVA results based on HALLMARK gene sets, ordered by t values. (B, C) GSEA-GO enrichment curves and ranked gene distributions for high-risk (B) and low-risk (C) groups. (D, E) GSEA-KEGG plots enrichment curves and ranked gene distributions for high-risk (D) and low-risk (E) groups.

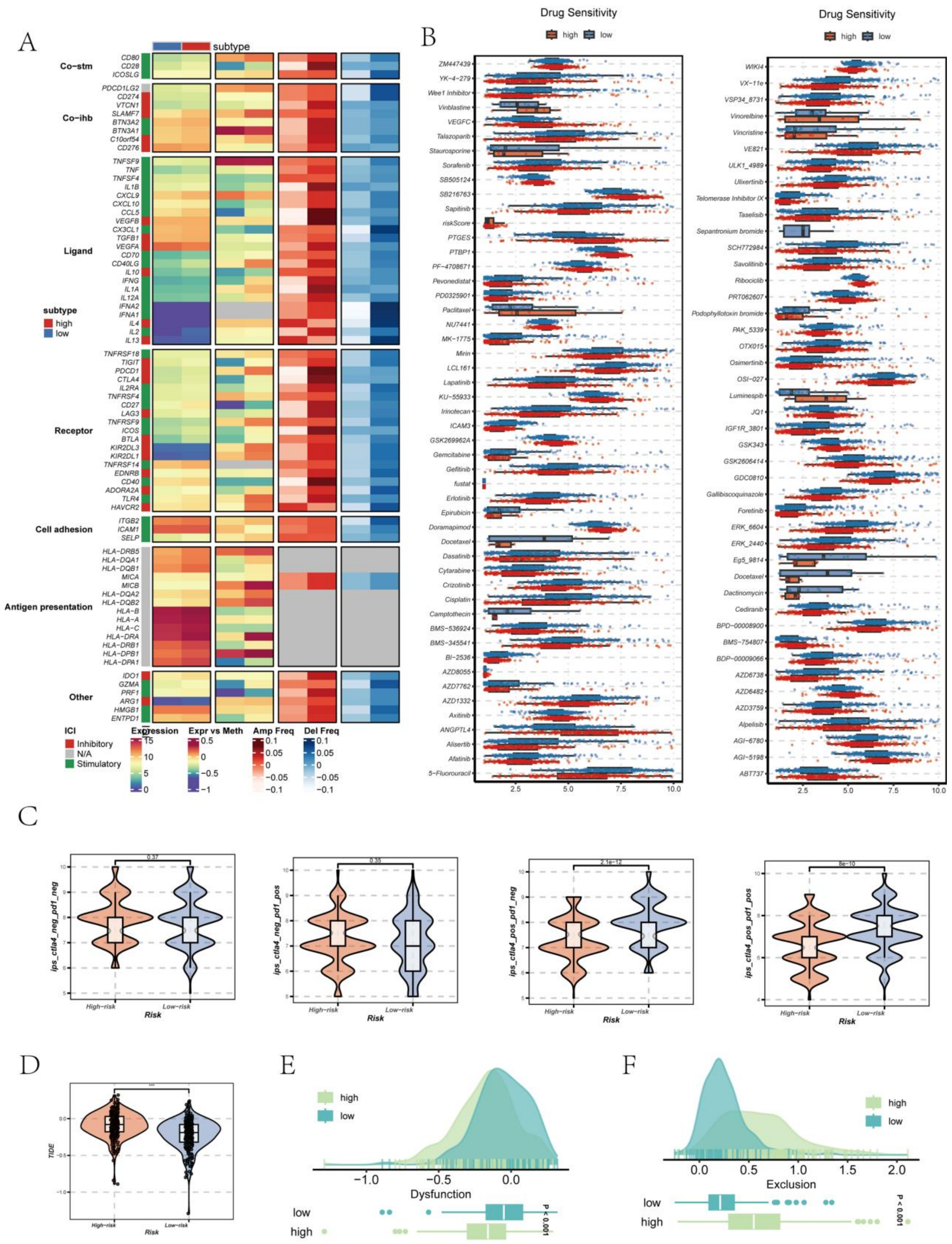

Figure S3. Overview of immune modulators, drug sensitivity, and immunotherapy-related indicators. (A) Heatmap of immune modulators across risk groups, including immune checkpoints, ligands, receptors, antigen-presentation molecules, and other immune-related regulators. (B) Distribution of predicted drug sensitivity (IC50) for multiple therapeutic agents in high- and low-risk groups. (C) Distribution of Immunophenoscore between the two risk groups. (D) Distribution of the overall TIDE scores. (E) Distribution of T cell dysfunction scores from the TIDE framework. (F) Distribution of T cell exclusion scores from the TIDE framework.

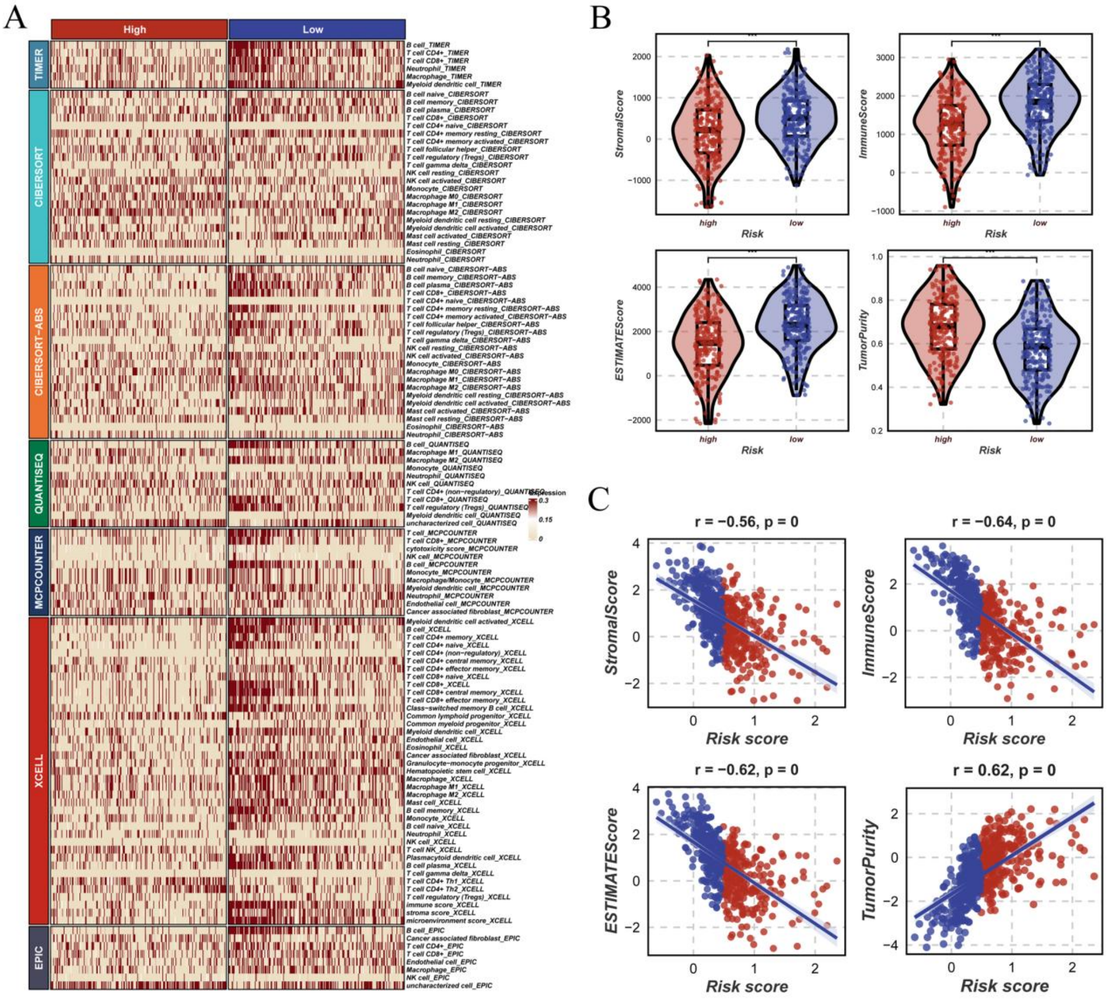

Figure S4. Immune infiltration profiles and ESTIMATE score evaluation. (A) Heatmap of immune cell infiltration across high- and low-risk groups using seven algorithms (TIMER, CIBERSORT, CIBERSORT-ABS, QUANTISEQ, MCPCOUNTER, XCELL, EPIC). (B) Distribution of ESTIMATE-derived scores, including StromalScore, ImmuneScore, ESTIMATEScore, and Tumor Purity. (C) Scatter plots showing correlations between the risk score and ESTIMATE metrics.

A

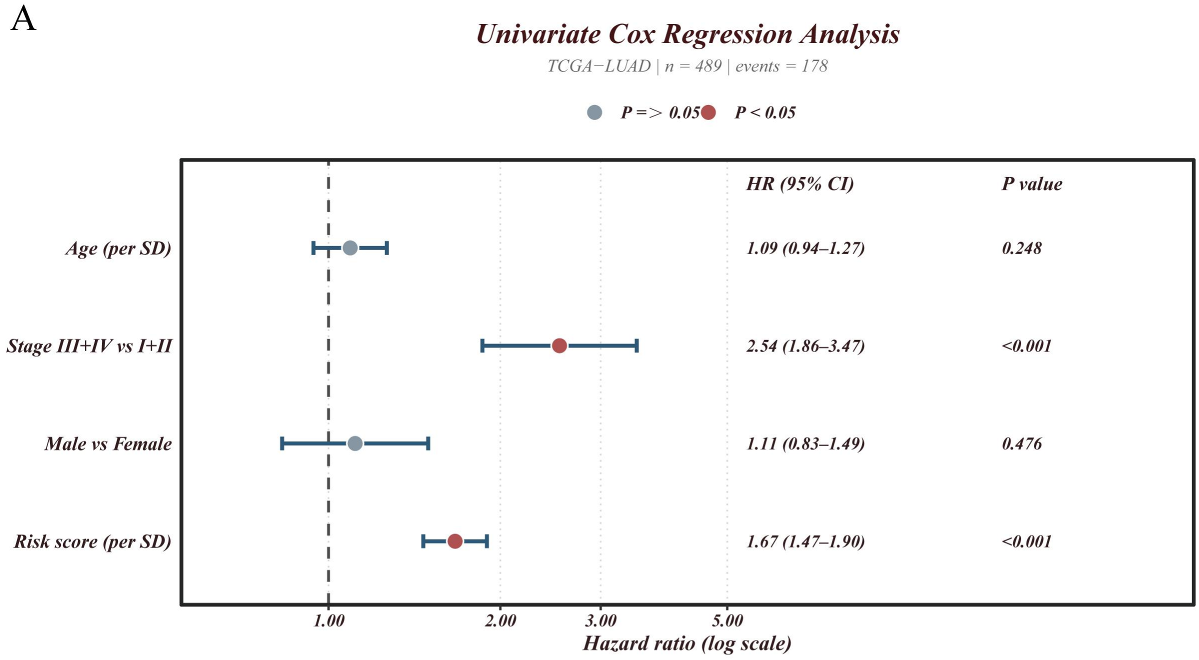

B

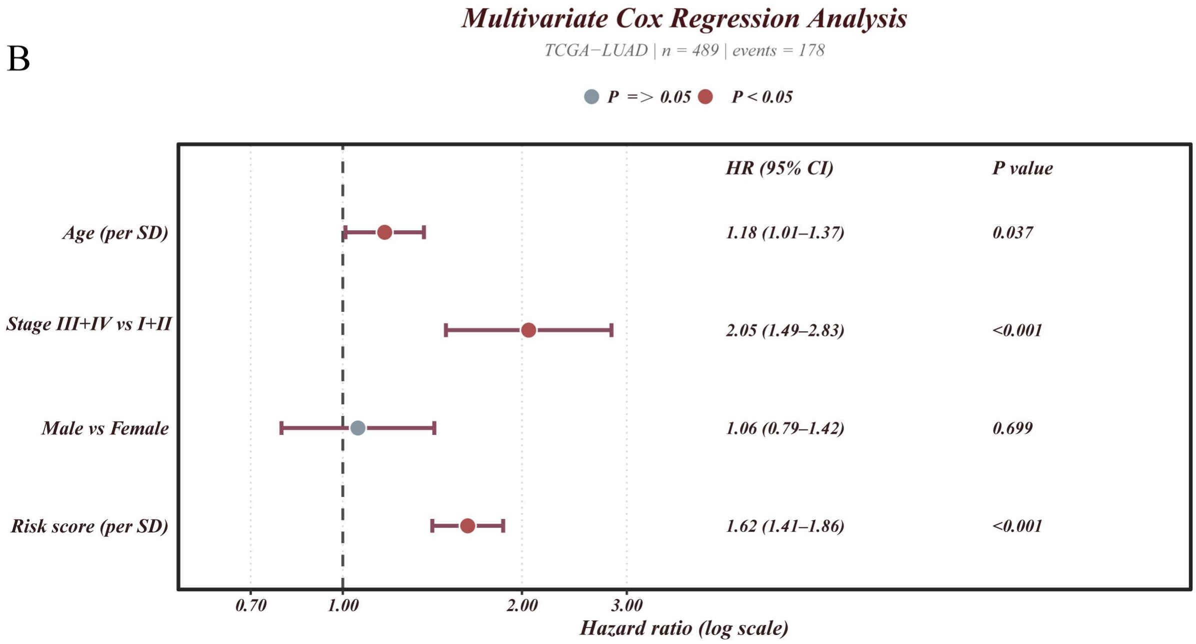

Supplementary Figure S5. Univariate (A) and multivariable (B) Cox analyses of overall survival in TCGA-LUAD. Analyses included 489 patients and 178 events. The multivariable model adjusted for age, pathological stage (III–IV versus I–II), sex (male versus female), and risk score. Hazard ratios for age and risk score are expressed per standard-deviation increase. Circles indicate hazard ratios and horizontal bars indicate 95% confidence intervals; the vertical dashed line denotes HR = 1. Blue indicates $p < 0.05$ and gray indicates $p \geq 0.05$.

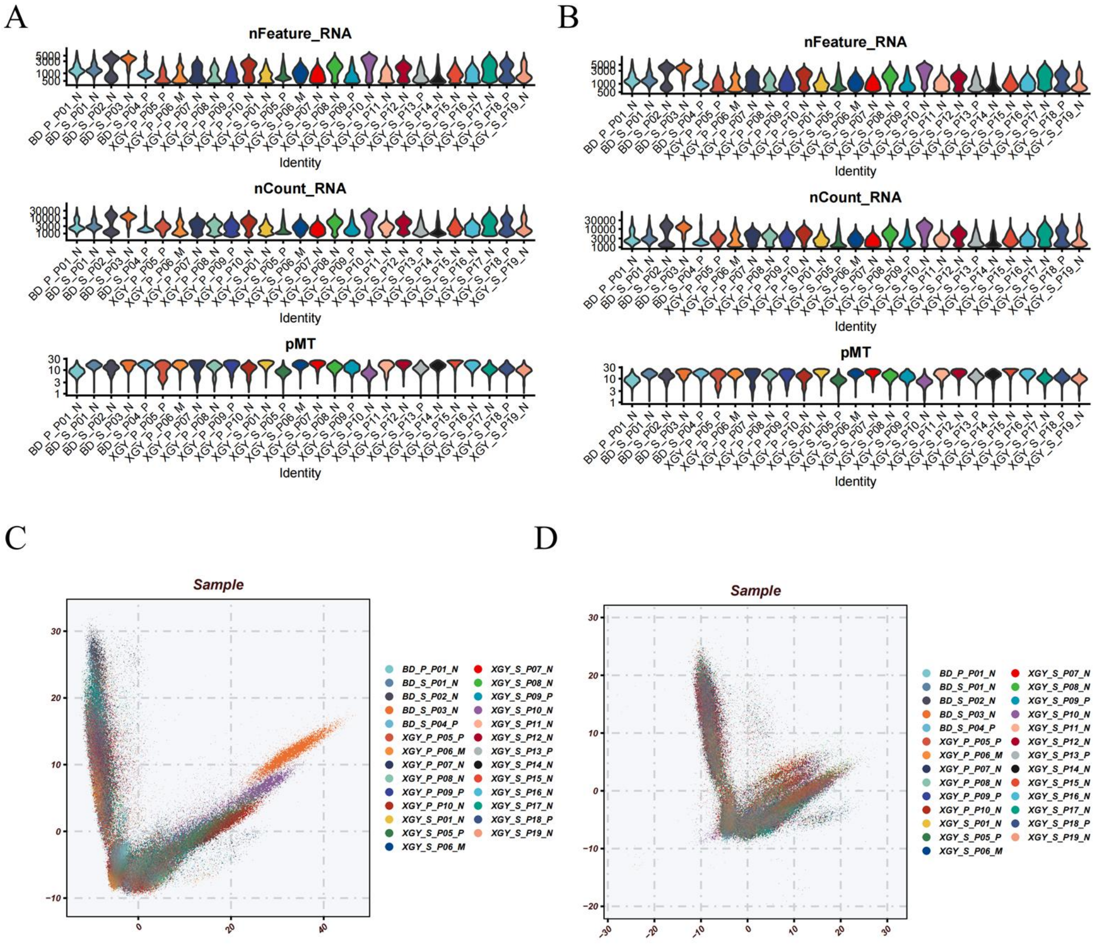

Figure S6. Quality control and batch-effect correction of single-cell data. (A) Distribution of nFeature_RNA, nCount_RNA, and mitochondrial gene percentage (pMT) across samples before quality control. (B) Distribution of nFeature_RNA, nCount_RNA, and pMT across samples after quality control. (C) UMAP projection of cells colored by sample origin before batch-effect correction. (D) UMAP projection of cells colored by sample origin after batch-effect correction using the Harmony algorithm

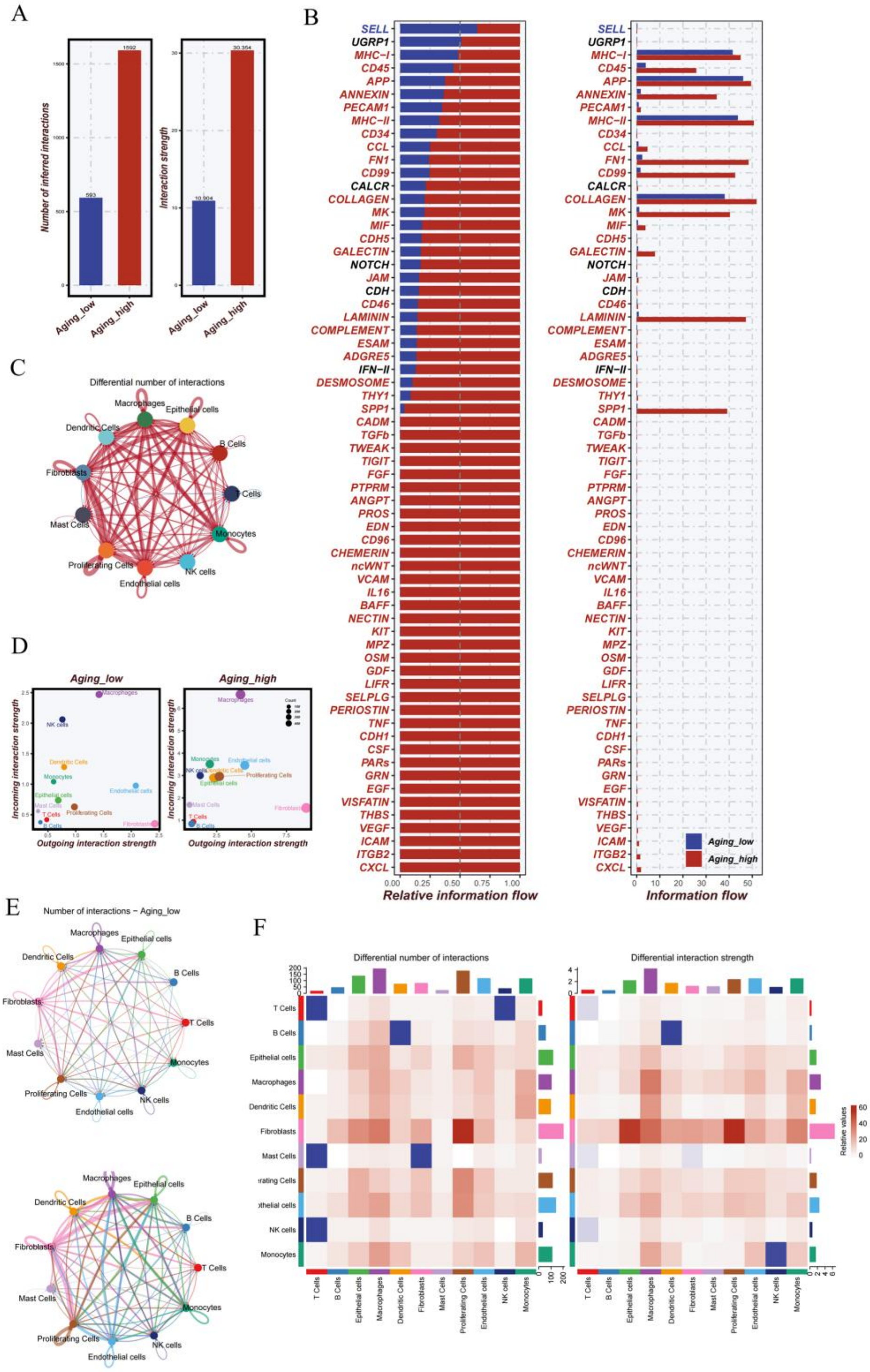

Figure S7. Cell-cell communication analysis between high- and low-senescence groups. (A) Comparison of the number of communication events and overall interaction strength. (B) Visualization of relative information flow and information flow strength for individual ligand–receptor pairs in both groups. (C) Cell-type-level interaction network illustrating the communication patterns among different cell populations. (D) Distribution of incoming and outgoing interaction strengths per cell type. (E) Composition of interacting cell types in low- versus high-senescence groups. F) Pairwise interaction frequencies between cell types.

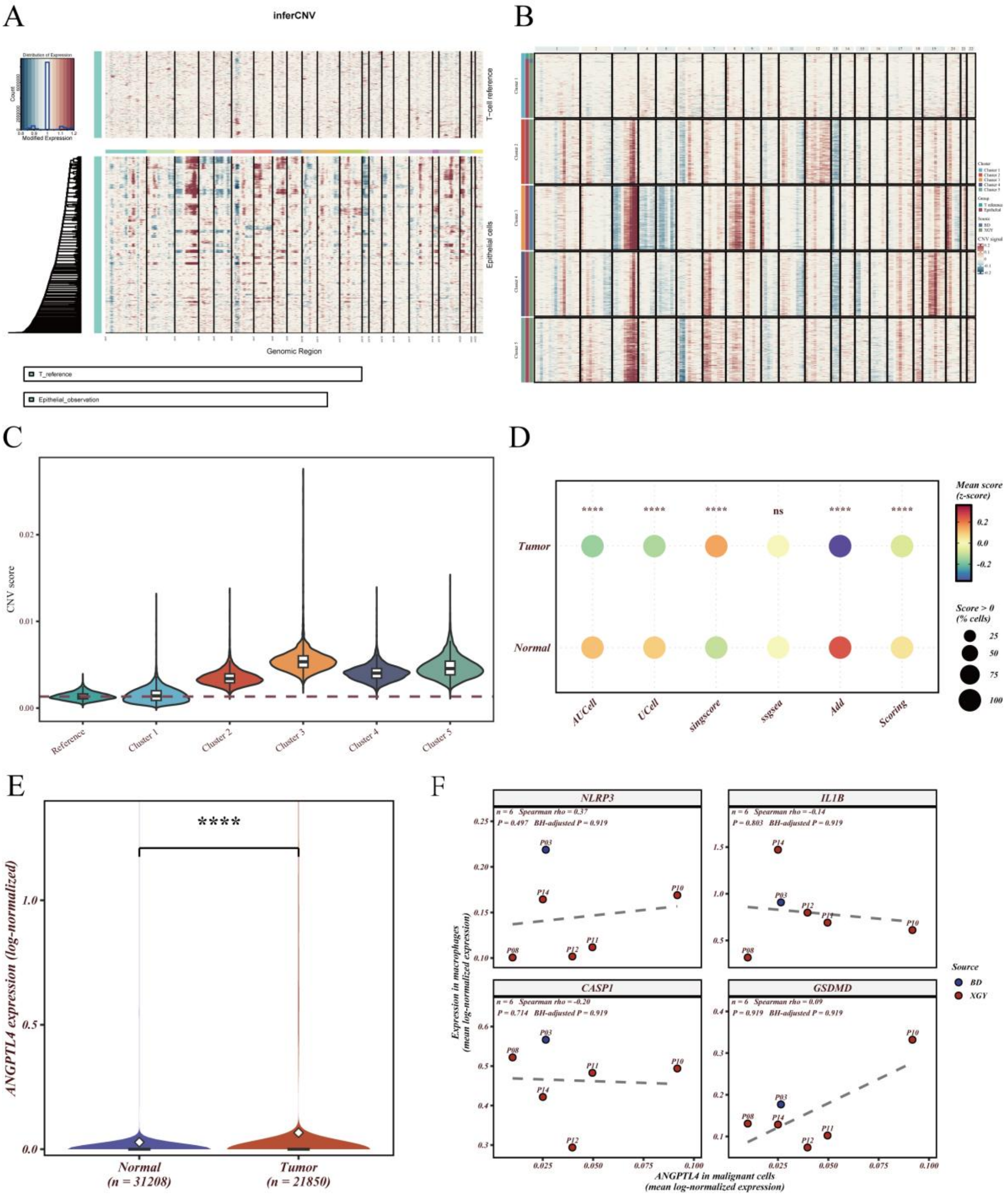

Supplementary Figure S8. Inferred epithelial malignancy and exploratory ANGPTL4 analyses. (A) Joint inferCNV analysis of epithelial cells using T cells as reference. (B) Five k-means clusters of CNV profiles. (C) CNV-score distributions in reference cells and epithelial clusters. Clusters 2–5 were classified as putatively malignant based on CNV patterns and scores. (D) Six senescence-associated score metrics in putatively nonmalignant (Normal) and malignant (Tumor) epithelial cells; color represents the group mean of standardized scores and point size the proportion with a score above zero. Wilcoxon tests were adjusted across metrics using Benjamini–Hochberg correction. (E) Log-normalized ANGPTL4 expression in 31,208 Normal and 21,850 Tumor epithelial cells. Diamonds indicate means. The upper display range is limited for readability; the cell-level Wilcoxon test includes all cells. In D–E, ****p < 0.0001; ns, not significant; D uses adjusted p-values. (F) Patient-level Spearman correlations between mean ANGPTL4 expression in

malignant cells and mean NLRP3, IL1B, CASP1, or GSDMD expression in macrophages. Each point is one postoperative patient with at least 30 cells of each type (n = 6). All four adjusted p-values are 0.919. The Normal label is an operational CNV-based classification, not independent confirmation of normal tissue.

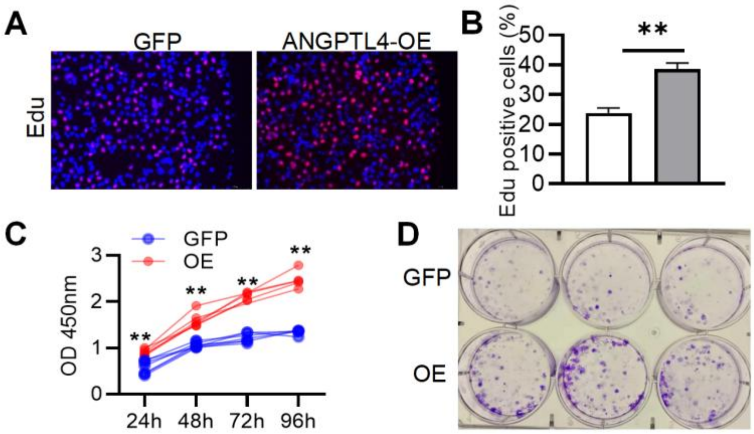

Figure S9. Overexpression of ANGPTL4 promotes proliferation of A549 cells. (A) Representative images of EdU staining. Scale bar, 100 µm. (B) Quantitative analysis of the proportion of EdU-positive cells (n=3). (C) the CCK- 8 assay (n=7). (D) Colony formation assay. Data are presented as mean ± SEM. *P < 0.05, **P < 0.01.

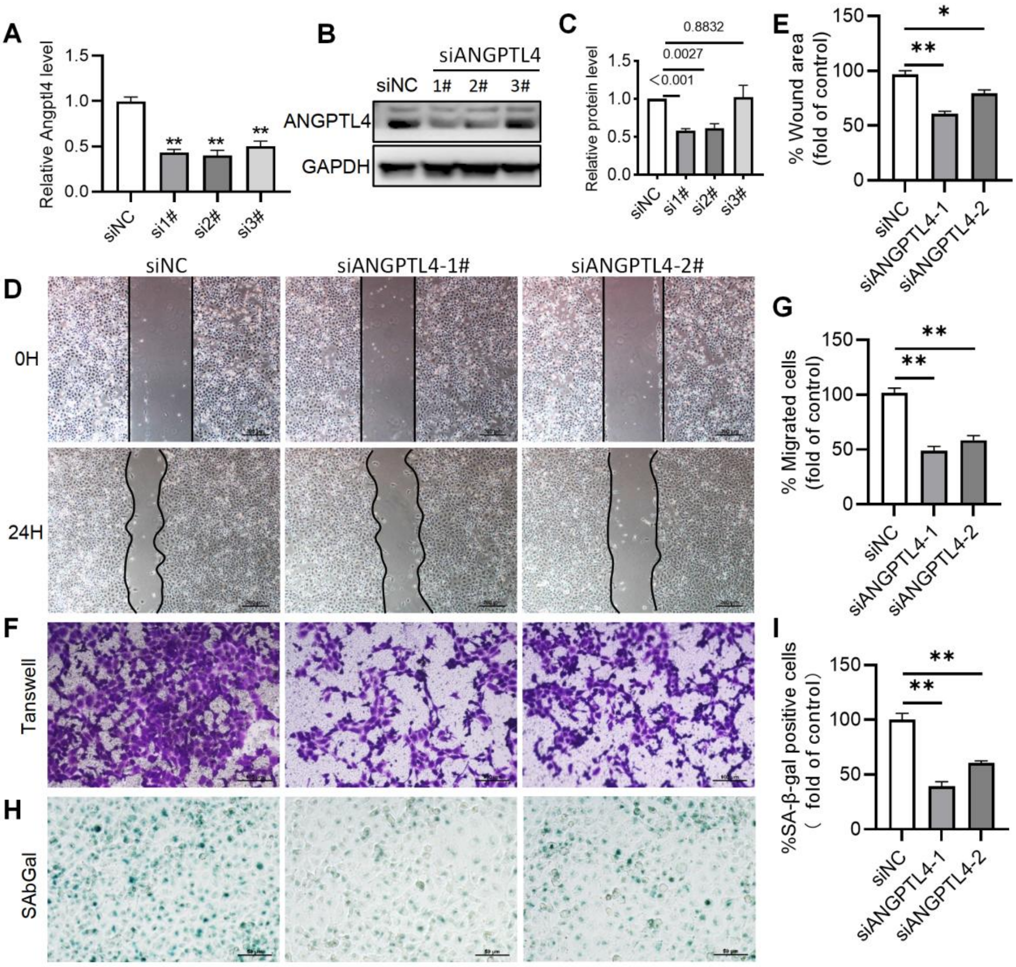

Figure S10. ANGPTL4 knockdown inhibits LUAD cell proliferation, migration, and SA-β-gal positivity in H1650 cells. (A) RT-PCR and (B, C) Western blot validation and qualification of ANGPTL4 knockdown efficiency in H1650 cells transfected with si-ANGPTL4 (n=3). (D and E) Wound healing assays showing impaired migratory ability after ANGPTL4 silencing. (F, G) Transwell migration assays demonstrating reduced migration following ANGPTL4 knockdown. (H, T) SA-β-gal staining showing a lower proportion of SA-β-gal-positive cells following ANGPTL4 depletion. Data are presented as mean ± SEM. *$P < 0.05$, **$P < 0.01$.

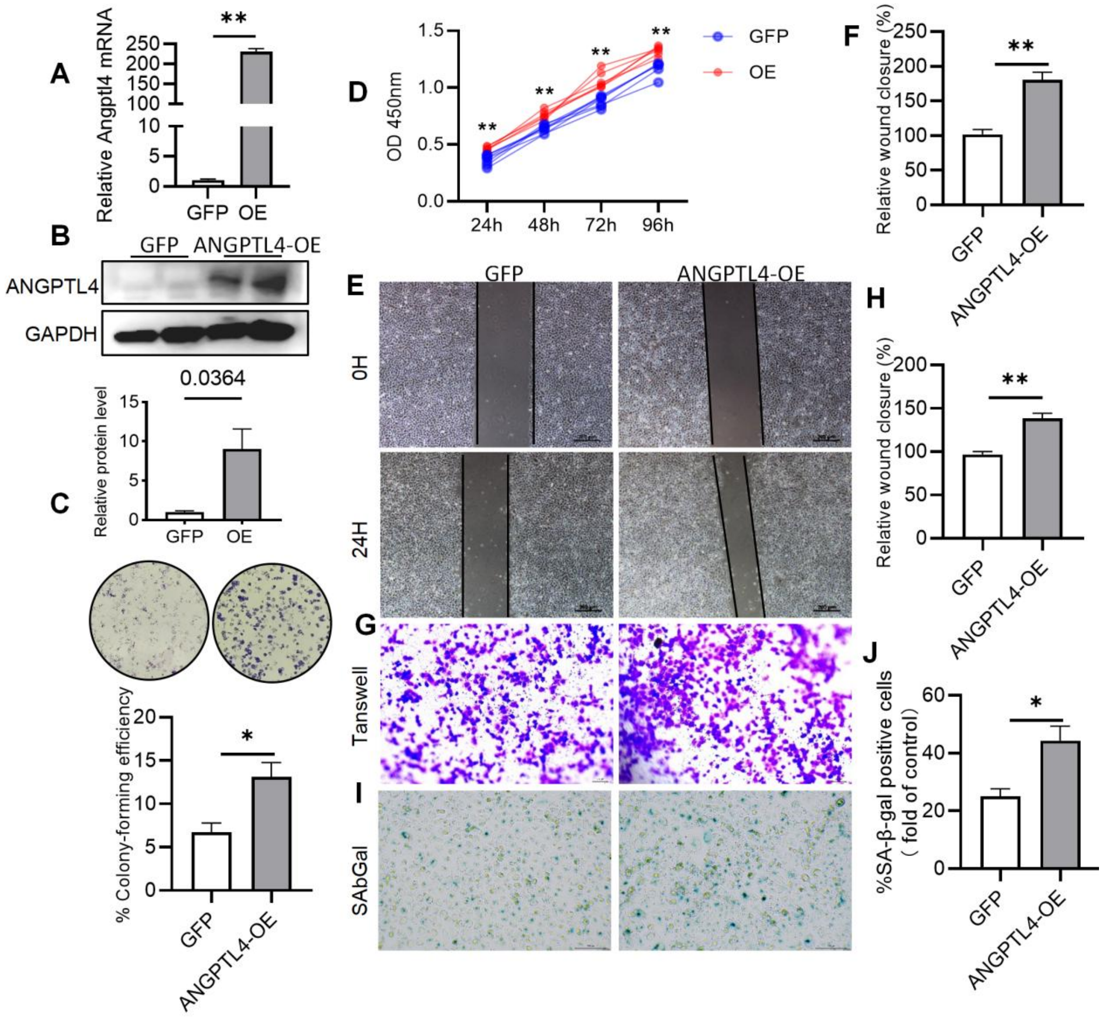

Figure S11. ANGPTL4 overexpression promotes LUAD cell proliferation, migration, and SA-β-gal positivity in H1650 cells.(A) RT-PCR and (B) Western blot validation (up pannel) and qualification (down pannel) of stable ANGPTL4 overexpression in H1650 cells (n=3). (C) Colony formation assays showing increased ability of colony formation in ANGPTL4-overexpressing cells. (D) CCK-8 assay showing slightly enhanced cell viability following ANGPTL4 overexpression. (E-F) Wound healing assays showing accelerated migration in ANGPTL4-overexpressing cells. (G-H) Transwell migration assays demonstrating increased migratory capacity. (I-J) SA-β-gal staining showing that ANGPTL4 overexpression also increases the SA-β-gal positivity. Data are presented as mean ± SEM. *$P < 0.05$, **$P < 0.01$.
